# Towards Intelligent Membrane Systems: A Critical Analysis of Digital Twin–ML Integration for Real–Time Process Optimization, Fouling Management, and Autonomous Design

**DOI:** 10.3390/membranes16100348

**Published:** 2026-10-20

**Authors:** Olawumi O. Sadare, Samuel P. Alonge, Azeez G. Akinyemi, Abass A. Adesina, Shukrah Adesola-Olabiyi, Lanrewaju I. Fajimi

**Affiliations:** 1 Department of Chemical and Bioprocess Engineering, School of Engineering and Innovation, Aston University, Aston Triangle, Birmingham B4 7ET, UK; 2 Department of Chemical Engineering Technology, University of Johannesburg, Johannesburg 2094, South Africa; proflonge1@gmail.com (S.P.A.); lanrefajimi@gmail.com (L.I.F.); 3 Department of Chemical Engineering, Northeastern University, Boston, MA 02115, USA; azeezakinyemi999@gmail.com; 4 Department of Electrical and Electronic Engineering Science, University of Johannesburg, Johannesburg 2094, South Africa; adesinaabassadedamola@gmail.com; 5 Department of Civil and Environmental Engineering, Tickle College of Engineering, University of Tennessee Knoxville, Knoxville, TN 37996, USA; olabiyibisolashukurat@gmail.com; 6 Postharvest and Agroprocessing Research Centre, Department of Botany and Plant Biotechnology, University of Johannesburg, PO Box 524, Auckland Park, Johannesburg 2006, South Africa

**Keywords:** digital twin, machine learning, membrane fouling, process optimization, autonomous design, physics-informed neural networks, reverse osmosis, membrane bioreactor

## Abstract

Membrane separation is central to potable water production, wastewater reclamation, desalination, gas separation, and decarbonization, yet its economics remain constrained by fouling and by a gap between process data and timely operating decisions. Digital twins (DTs) offer live, bidirectionally coupled virtual representations of physical assets, while machine learning (ML) extracts predictive and prescriptive insights from operational data. Together, they are promising computational approaches for membrane systems. However, their applications have largely evolved independently across fouling prediction, DT development, and ML-based materials discovery. This review argues that these are converging strands of a single trajectory toward self-sensing, self-predicting, and eventually self-designing membrane systems, and that treating them separately obscures key interdependencies. The contributions are threefold. First, applying the digital model/digital shadow/digital twin taxonomy, it shows that most published membrane DTs are digital shadows (DSs). Second, it proposes a five-level autonomy framework, inspired by vehicle automation taxonomies, providing a unified scale for comparing applications ranging from LSTM-based fouling forecasting and reinforcement learning dosing control to autonomous materials laboratories. Third, it integrates autonomous materials design, including self-driving laboratories, inverse design, generative models, and Bayesian search, into the upper levels of this ladder. Evidence is synthesized across reverse osmosis, nanofiltration, ultrafiltration, microfiltration, membrane bioreactors, membrane distillation, and gas separation membranes, with emphasis on critically assessing model performance and generalizability. The study concludes with a roadmap for overcoming data limitations, poor interpretability, interoperability gaps, cybersecurity risks, techno-economic barriers, and regulatory trust deficits that block autonomous membrane systems.

## 1. Introduction

Membrane processes now occupy a central position in the world’s water, energy, and decarbonization infrastructure, underpinned by decades of materials innovation and process engineering advances [1]. Reverse osmosis (RO) desalinates seawater and brackish groundwater at a scale that would have been unthinkable three decades ago, with global installed RO capacity exceeding 100 million m^3^/day and continuing to grow driven by freshwater scarcity and falling energy costs [2]. Membrane bioreactors (MBRs) have transitioned from a niche technology to a mainstream wastewater reclamation solution deployed on every inhabited continent, with the global MBR market projected to surpass USD 9 billion by 2034 [3]. Nanofiltration (NF) and ultrafiltration (UF) are now embedded within municipal drinking water treatment trains and industrial separation processes, offering selective ion rejection and pathogen removal at significantly lower energy demand than thermal alternatives [4]. Membrane distillation (MD), pervaporation, and polymeric gas separation membranes are being actively advanced as low-energy routes to carbon capture, hydrogen purification, and solvent recovery [5,6]. These developments position membrane technology as a critical enabler of industrial decarbonization [7].

Despite this diversity of application, these processes are governed by several common physical constraints. A semi-permeable membrane separates two streams under a driving force while rejected materials accumulate at or within the membrane [8]. This accumulation reduces membrane flux and increases operating pressure and energy demand [9]. Ultimately, severe membrane degradation can force the process offline for chemical cleaning or membrane replacement. Fouling is not merely a peripheral operational nuisance; it is often the primary limiting factor in membrane plant design, operating costs, and membrane lifetime [9,10]. Its behavior is strongly influenced by feedwater variability, pretreatment performance, and cleaning, creating dynamic interactions that classical static process models struggle to capture [10].

Historically, fouling management in membrane systems has relied largely on reactive interventions, including predetermined cleaning cycles, empirically derived fouling indices, and operator experience [9]. These approaches have often substituted for the predictive insight needed to anticipate fouling development and enable proactive control of membrane performance degradation. Real-time monitoring and predictive modeling are increasingly being developed to address this limitation [11]. The past decade has produced two distinct and largely separately narrated responses to this gap.

The first is the application of machine learning (ML) to fouling and performance prediction. For example, artificial neural network (ANN) models have been applied to relate temperature, transmembrane pressure (TMP), and operating time to permeate flux and fouling resistance [12]. This demonstrates their potential for accurately forecasting membrane performance under varying operating conditions. Beyond ANN-based approaches, support vector regression (SVR), linear regression (LR), and decision-tree regression (DTR) have been applied to predict TMP in MBRs [13]. Similarly, Kovacs et al. [14] compared random forest (RF), ANN, and long short-term memory network (LSTM) models using four years of high-resolution data from a full-scale municipal MBR. The study concluded that all three ML models provided reliable TMP predictions.

The second is the emergence of digital twins (DTs) as a bridge between data-driven prediction and intelligent membrane operation. In membrane systems, a DT combines a continuously updated virtual representation of the membrane process with real-time sensor and plant data, enabling dynamic monitoring, performance forecasting, fouling diagnosis, and scenario-based optimization [15]. Zhu et al. [16] demonstrated ML-enabled DT frameworks for MBRs, where time series models forecast fouling and permeability while newer DT architectures extend these predictions toward proactive cleaning and process-control decisions [15]. Such developments shift membrane management from reactive intervention toward predictive, adaptive, and potentially autonomous operation.

A third and increasingly important thread moves ML beyond the operation of existing membranes toward the inverse design and discovery of new membrane materials. Rather than predicting the performance of a predefined membrane, these approaches learn structure–property relationships and then search chemical or polymer design spaces for materials with targeted permeability and selectivity [17]. Recent ML-guided genetic Bayesian and generative design frameworks have identified candidate polymers predicted to exceed established Robeson upper bounds for CO_2_/N_2_ and CO_2_/CH_4_ separations, with some candidates subsequently evaluated using molecular dynamics simulations [18,19]. This paradigm transforms ML from a predictive tool into a mechanism for accelerated membrane discovery and materials-by-design.

This review critically examines the integration of DTs and ML across membrane systems, spanning real-time process optimization, fouling management, and autonomous membrane design. Unlike existing reviews that largely treat fouling prediction, DT-enabled process operation, and ML-assisted materials discovery as separate research domains, this work develops a unified framework linking these applications along a common autonomy continuum. The review distinguishes digital models (DMs), digital shadows (DSs), and true DTs based on the direction and fidelity of data exchange while positioning ML according to its function within the DT loop, which varies from state estimation and prediction to diagnosis and prescriptive control. It further extends the autonomy framework from membrane operation to inverse design and self-driving materials discovery, treating autonomous design as a higher level of the same digital intelligence continuum. By integrating these traditionally disconnected domains, the review identifies maturity gaps, technological barriers, and pathways toward a closed design–operate–maintain ecosystem for intelligent membrane systems.

## 2. Digital Twins and Machine Learning in Membrane Systems

### 2.1. Digital Twin (DT)

**Figure 1:**
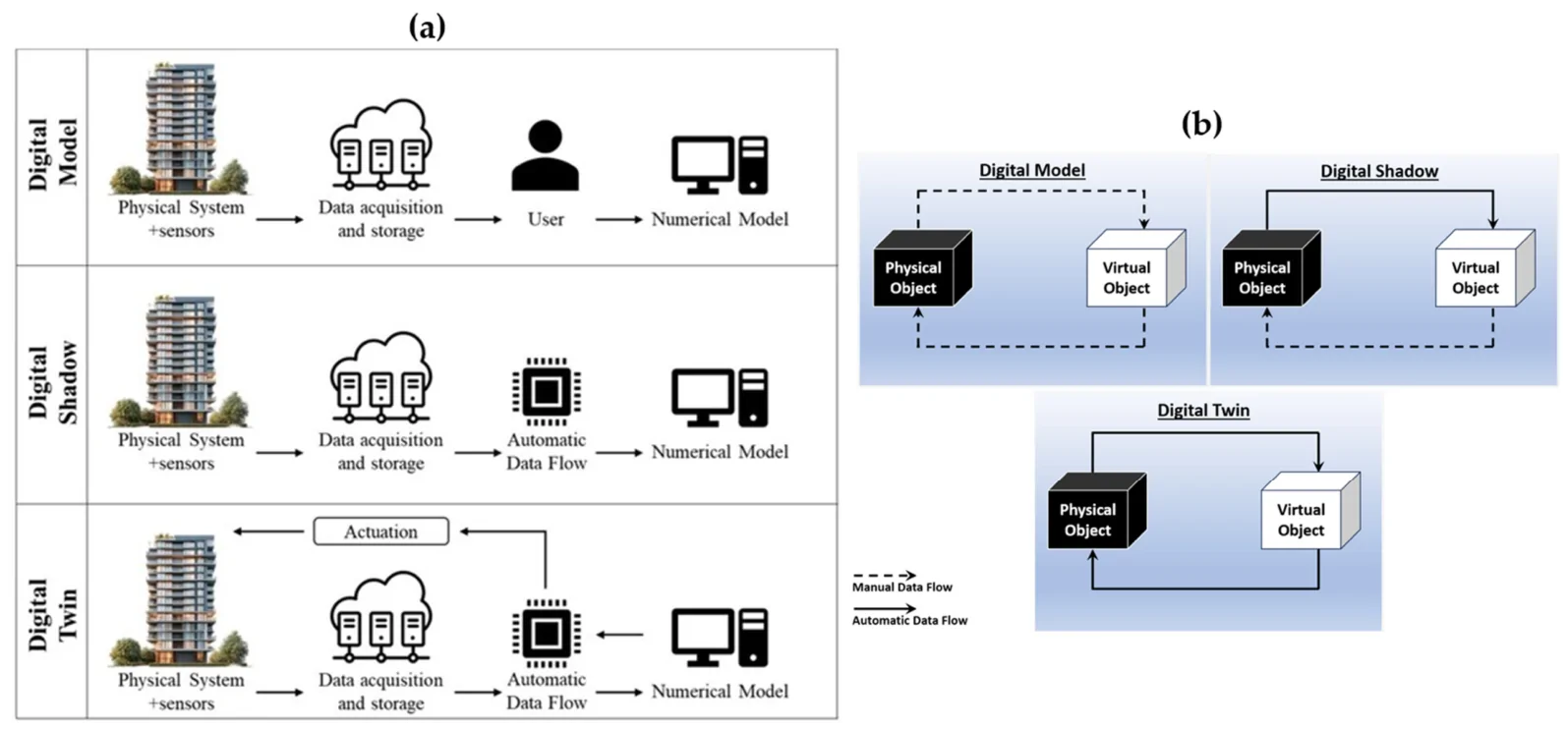
Digital model, digital shadow, and digital twin: (**a**) illustration [22]; (**b**) classification according to the direction of data integration. Adapted from [28].

The term DT is increasingly used across membrane and water treatment research, but its meaning varies considerably. A useful foundation is the framework of Grieves and Vickers [20] and the subsequent classification by Kritzinger et al. [21], which distinguishes digital representations according to their degree of integration with the physical system (Figure 1). A digital model (DM) is a virtual representation based on design information, empirical relationships, or mechanistic equations without automated data exchange with the physical asset [21]. A digital shadow (DS) introduces automated, one-way data transfer from the physical system to the virtual representation, allowing the latter to track changing operating conditions [22]. A DT, in contrast, incorporates automated bidirectional data exchange, allowing the virtual system to not only receive information from the physical asset but to also communicate control or intervention information back to it [21,23]. This distinction is particularly important for membrane systems. A model that predicts transmembrane pressure (TMP), permeate flux, or fouling from historical operating data is not necessarily a DT. Similarly, an ML-based fouling predictor that continuously receives supervisory control and data acquisition (SCADA) data but only presents recommendations to an operator is more appropriately considered a DS or decision support system [24]. A genuinely integrated DT would use continuously updated process information to estimate membrane state and subsequently feed-optimized operating actions such as flux, pressure, recovery, chemical dosing, or cleaning decisions back to the physical process. Such applications are beginning to emerge in membrane technology. For example, a DT-based framework has been developed to evaluate membrane restoration strategies in RO desalination using degradation states of individual membrane elements while recent work has explored DT–ML integration for real-time seawater reverse osmosis (SWRO) efficiency management [25]. Figure 1a should therefore emphasize the data flow distinction rather than simply presenting three levels of modeling sophistication, as illustrated in Figure 1b. Beyond connectivity, DTs differ in model fidelity, synchronization frequency, computational architecture, and lifecycle role. Underlying models range from ML surrogates and reduced-order mechanistic models to high-fidelity computational fluid dynamics (CFD) and multiphysics simulation, and implementations range from laboratory and pilot representations to plants connected through sensors, Internet of Things (IoT) infrastructure, and SCADA [26,27]. SCADA integration alone does not make a DT: if information flows only from plant to virtual environment and decisions remain human-mediated, the system remains a DS. This distinction underpins the assessment of autonomy throughout the review.

### 2.2. A Functional Taxonomy of ML in Membrane Systems

**Figure 2:**
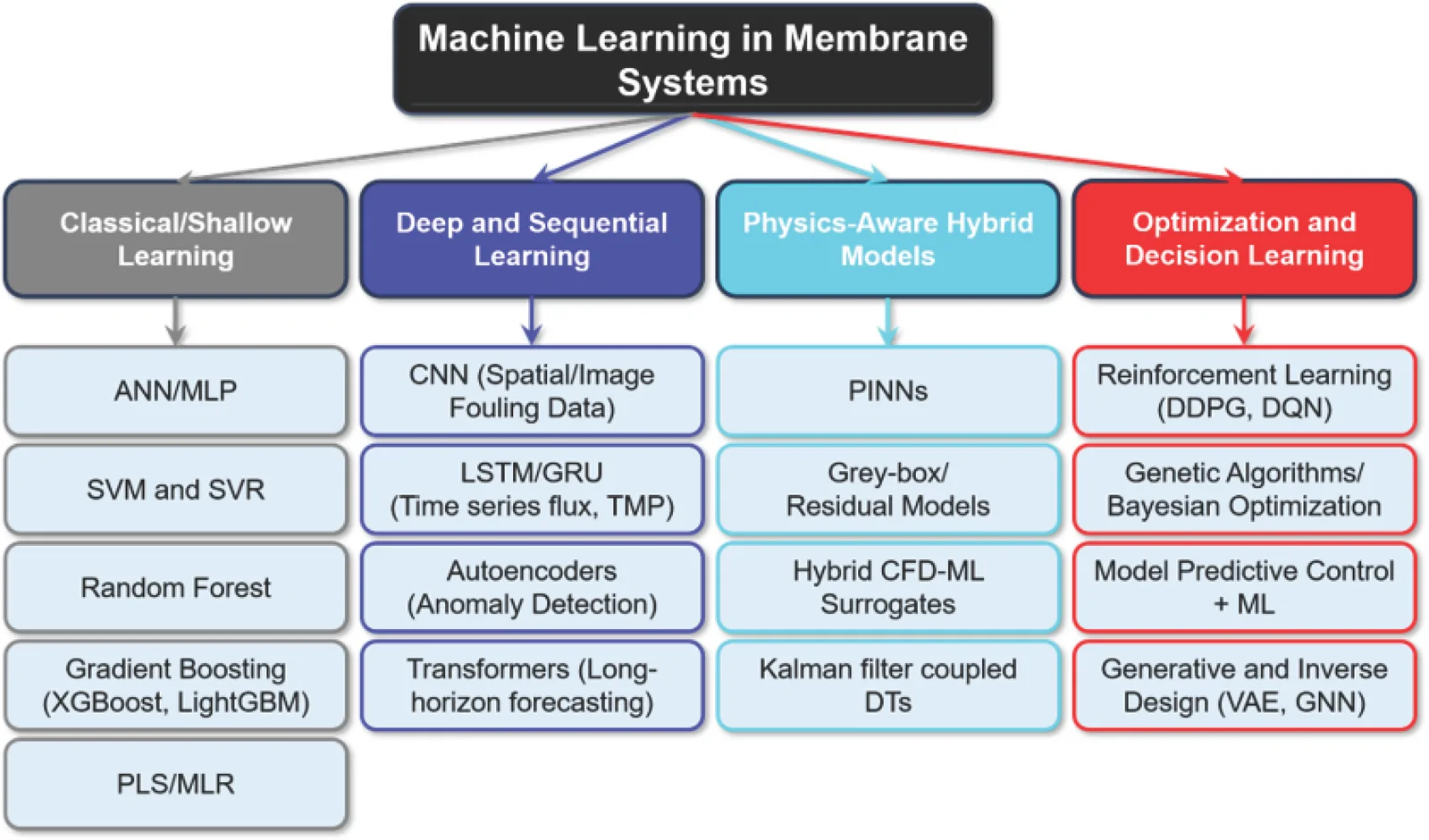
Taxonomy of ML approaches applied to membrane systems organized by function within the DT loop.

Existing AI/ML reviews classify studies by algorithm family (ANN, SVM, random forest, and so on). This is useful for comparing techniques but says little about where a model sits on the DM–DS–DT continuum or what operational function it serves; prediction alone does not constitute a DT. This study therefore adopts a functional taxonomy (Figure 2) with four bands. These bands correspond to the prediction/reasoning and decision/actuation layers of the architecture in Section 4. An ML forecast does not convert a DS into a DT; the decisive transition occurs when the virtual representation is continuously coupled to the physical system and can influence it through an automated feedback pathway [21]. Prediction supplies intelligence; validated closed-loop decision and actuation supply autonomy.

Classical and shallow learning (ANN, SVR, random forest, gradient boosting such as XGBoost and LightGBM, partial least squares, and multiple linear regression) work with small-to-moderate datasets and modest computation. Full-scale RO studies show that ANNs can distinguish temporally different fouling regimes and identify variables linked to particle deposition, biological growth, and scaling [29,30], and conventional ML remains central to fouling diagnosis and prediction [11,31,32]. Site-specific metrics, however, are not universal benchmarks because dataset size, temporal dependence, feature availability, and validation strategy strongly shape reported accuracy.

Deep and sequence learning suit strong temporal or spatial dependence. Convolutional neural networks (CNNs) handle image- or spatially structured data [33], while LSTM and related recurrent models capture temporal dependence in flux [34], TMP, permeability, and fouling trajectories. A full-scale MBR sequence-to-sequence/LSTM model reported MAPE below 6.5% and R^2^ > 0.87 [16]. High forecasting accuracy, however, is not autonomy: the model remains a predictive component until its outputs connect to a validated decision and control architecture.

Physics-aware and hybrid learning is especially important for DTs because it combines ML adaptability with mechanistic knowledge: grey-box and residual models, physics-informed neural networks (PINNs), CFD–ML surrogates, and state estimators [35,36]. Raissi et al. [37] established the PINN framework; Gaublomme et al. [38] coupled an LSTM fouling model with a solution–diffusion RO model and improved long-horizon prediction over the mechanistic model alone; and Møller et al. [39] built a stochastic grey-box UF DT that represented uncertainty explicitly and supported real-time control.

Optimization and decision learning is the prescriptive layer: reinforcement learning (RL), Bayesian optimization (BO), genetic algorithms (GAs), model-predictive control (MPC), and ML-based inverse design determine setpoints, maintenance actions, or candidate structures. Heusel et al. [40] embedded a regression metamodel in an industrial filtration DT for near-real-time optimization; in materials research, forward models coupled to GAs identify polymers meeting separation targets [41,42]; and autonomous laboratories close the experimental loop through automated synthesis, characterization, and ML-guided experiment selection [43].

It should be noted that the proposed taxonomy is functional rather than mutually exclusive, as a single ML architecture may simultaneously perform prediction, optimization, and control. Classification is therefore based on the primary role of the ML component within the membrane decision loop: prediction estimates future process states or outcomes, optimization identifies preferred operating decisions under specified objectives and constraints, while control translates process information into operational actions. Hybrid models are classified according to their dominant operational function, while their complementary predictive, optimization, or control capabilities are also acknowledged.

### 2.3. Explainability as a Cross-Cutting Requirement

As ML moves from informing to influencing operational decisions, accuracy alone is insufficient. Operators need to know why a prediction was made, which variables drive it, and whether the relationships agree with membrane process knowledge; opacity undermines trust, accountability, and regulatory acceptance, and explainability is recognized as a component of trustworthy, human-in-the-loop, safety-critical DTs [44]. SHAP (Shapley Additive Explanations) and LIME (Local Interpretable Model-Agnostic Explanations) are complementary tools [45]. SHAP quantifies the contribution of individual inputs to model outputs, supporting both global feature ranking and case-level interpretation, while LIME constructs local surrogate explanations but can be sensitive to sampling and neighborhood selection [45]. In membrane systems, however, the value of XAI extends beyond identifying statistically important variables: explanations should be interpreted against established fouling and transport mechanisms. For example, SHAP-based analysis of RO flux predictions has shown the importance of feed flow rate and applied pressure [46], while XAI-based MBR studies have identified operational and biological variables such as F/M ratio and mixed liquor suspended solids as influential predictors of fouling [47]. Such relationships can be interpreted in terms of hydraulic loading, boundary-layer development, particulate/organic deposition, and changes in fouling resistance. Similarly, in forward osmosis, SHAP analysis has identified osmotic pressure difference and water permeability as dominant contributors to flux prediction, with the resulting interpretations shown to be consistent with physicochemical modeling. This illustrates how XAI can connect data-driven predictions to membrane transport principles rather than merely rank model inputs [48]. Accordingly, explanations should be linked to physical mechanisms, operating constraints, and uncertainty, enabling DTs to distinguish physically meaningful signals from spurious correlations. As decision authority shifts from human operators toward automated intervention, XAI should therefore be coupled with uncertainty quantification, physics-based constraints, and audit trails; models that cannot provide interpretable, mechanism-consistent justification may remain useful for monitoring but are less suitable for autonomous membrane process control [49].

## 3. How This Review Differs from the Existing Literature

### 3.1. Literature-Search Strategy and Thematic Classification

To establish the position of the present review within the rapidly expanding literature on membrane intelligence, an extensive literature search was conducted across Scopus, Web of Science (WoS), and Google Scholar, with emphasis on publications from January 2019 to date. The search focused on the intersection of membrane technology, machine learning (ML), digital models (DMs), digital shadows (DSs), digital twins (DTs), process optimization, fouling management, autonomous control, inverse design, and materials discovery. Particular attention was given to review articles because the purpose of this section is to establish the existing review landscape and identify areas that have already been comprehensively synthesized. Four thematic search clusters (see Table 1) were defined a priori to provide a structured basis for classifying the retrieved literature.

The search strategy produced 73 Scopus retrievals across the four clusters (C1–C4: 16, 13, 3, and 41), 31 WoS retrievals (5, 0, 6, and 20), and 1827 Google Scholar retrievals (62, 748, 128, and 889), giving 1931 database-by-cluster retrievals in total. These values represent database retrievals rather than unique publications because the same record may occur across databases and may also be retrieved by more than one thematic search. Google Scholar was therefore used as a complementary source rather than treated as directly equivalent to the structured indexing of Scopus and WoS. Records were exported, de-duplicated by DOI and normalized title, and screened against the following criteria: [inclusion: review articles addressing AI/ML, hybrid modeling, DM/DS/DT, or ML-assisted design of membranes or membrane-based water treatment; exclusion: original research, non-English records, and reviews in which membranes or AI/ML were not a substantive focus].

Cluster 1: AI/ML-assisted membrane fouling prediction and control: The body of review literature focuses on the application of AI and ML to membrane fouling, performance prediction, diagnosis, and control. Representative reviews include those by Bagheri et al. [11] and subsequent studies covering ANNs, SVMs, ensemble learning, deep learning, and other data-driven approaches [11,31,32,50,51,52,53,54,55,56,57,58,59,60,61,62]. These reviews provide valuable comparisons of algorithms, input variables, fouling indicators, and reported predictive performance. The distinction between prediction, decision support, and closed-loop autonomy is rarely the organizing principle, and links to materials discovery are outside scope.

Cluster 2: DTs in the water and wastewater sector: The second cluster treats DTs as a digitalization and infrastructure paradigm: sensing, IoT, monitoring, hybrid modeling, predictive maintenance, standards, and adoption [24,27,63,64,65]. Membrane-specific fouling mechanisms and ML architectures get less attention, materials design is absent, and the literature illustrates the terminological problem of Section 2: systems called DTs differ greatly in automated interaction with the process.

Cluster 3: Hybrid modeling, DSs, and DTs for membrane systems: The third cluster is most closely aligned with the operational focus of the present review. These studies examine hybrid mechanistic–data-driven modeling, digital representations, and DS/DT architectures and their application to membrane processes, including water treatment, gas separation, and emerging membrane applications [36,45,66,67]. By adding process physics, CFD, and state estimation, it addresses the limits of purely empirical ML and supplies the technical basis for Section 4, but it emphasizes modeling architecture, not autonomous materials discovery or self-driving laboratories.

Cluster 4: ML-assisted membrane materials design and discovery: The fourth cluster covers property prediction, structure–property relationships, forward and inverse design, and candidate discovery [17,41,42,68,69,70,71,72,73,74,75,76,77,78], extending toward BO, GAs, generative models, and autonomous platforms [43,70]. It has developed largely apart from operational DTs: plant-level fouling, degradation, cleaning, and lifetime data rarely inform material discovery, and new materials are seldom evaluated in an operational DT.

### 3.2. The Unresolved Gap and Position of the Present Review Relative to Previous Reviews

The central gap identified by this review is not a lack of ML algorithms, DT architecture, or materials informatics methods; rather, it is the absence of a common framework for understanding how these capabilities combine to reduce human intervention across the membrane lifecycle. Fouling prediction work asks whether a model can estimate flux decline or TMP; DT work connects assets, sensors, and models; and materials informatics treats development as separate from plant operation. This was addressed through three linked contributions. First, this review applies a consistent DM–DS–DT taxonomy, judging the direction of information exchange rather than adopting each paper’s terminology. Second, this review introduces an autonomy framework (Levels 0–5) describing how much decision-making authority is delegated to the intelligent system; it is distinct from the DM–DS–DT taxonomy, so a genuine DT can sit at a low autonomy level if humans retain authority over substantive interventions. Third, this review connects operational intelligence with ML-assisted materials discovery, treating autonomous design as the upper stage of the same continuum, in which operational DTs set materials requirements and new materials are evaluated in process-level digital environments. This is a cross-domain synthesis rather than another algorithm survey (Table 2); Section 4, Section 5, Section 6, Section 7, Section 8 and Section 9 build the architecture and evidence, Section 10 formalizes the framework, Section 11 applies it across technologies, and Section 12 and Section 13 address barriers and the roadmap.

## 4. A Reference Architecture for Intelligent Membrane Digital Twins

### 4.1. Five-Layer Reference Architecture

Published membrane DTs differ substantially in scope, ranging from individual ML models for membrane performance or fouling prediction to more comprehensive DT platforms integrating process models, real-time data, monitoring, and control. DT reviews similarly describe architectures that combine physical assets and sensors with data infrastructure, mechanistic/data-driven models, IoT connectivity, and control functions [24,36,65]. Despite these differences, their architecture shares several common components and functional layers.

Layer 1 (Physical and Sensing) comprises the membrane assets themselves, including RO pressure vessels, UF/MF racks, MBR tanks, NF skids, and spiral-wound or hollow-fiber modules. These systems are equipped with sensors for TMP, permeate flux, and water quality/process variables, while online ultrasonic techniques have also been demonstrated for non-invasive monitoring of membrane fouling and fouling-layer development [79]. Data are transmitted through SCADA, programmable logic controllers (PLCs), and edge-IoT devices to support real-time monitoring [24].

Layer 2 (Data Integration and Contextualization) transforms raw sensor streams into reliable, analysis-ready data by addressing missing values, timestamp inconsistencies, sensor failures, and planned shutdowns. This layer incorporates data imputation techniques, temporal synchronization across historian and SCADA systems, and feature engineering to derive fouling-relevant indicators such as rates of change, rolling statistics, and fouling indices. Recent full-scale MBR work has used moving average features and operational variables to capture temporal fouling behavior and improve predictive modeling [47].

Layer 3 (Hybrid Modeling) integrates mechanistic, physics-based, and data-driven approaches to provide a physically consistent representation of membrane behavior. Hybrid membrane modeling increasingly combines physical equations with ML models, while CFD-generated data can be used to develop computationally efficient surrogate models [36]. It incorporates solution–diffusion and resistance-in-series formulations, CFD-derived surrogate models, and physics-informed neural networks (PINNs), which constrain ML predictions using governing physical relationships. The solution–diffusion model remains a fundamental framework for describing transport through dense membranes, while resistance-in-series formulations are widely used to represent the contributions of different fouling resistances [35,45].

Layer 4 (Cognitive/ML Reasoning) operationalizes the functional ML taxonomy introduced in Section 2.2 by transforming process data into actionable information, including fouling and remaining-useful-life (RUL) forecasting, anomaly detection, state estimation, and predictive performance assessment. Recent studies demonstrate ML-based prediction of membrane fouling, transmembrane pressure, permeate flux, and RUL, including the use of SHAP-based analyses to identify influential operational variables [47,80].

Layer 5 (Decision and Actuation) converts model outputs into operational actions, such as setpoint adjustments, chemical-dosing changes, cleaning-in-place (CIP) triggers, maintenance scheduling, or membrane replacement decisions. DT reviews of wastewater systems identify optimization, predictive maintenance, and control as important functions of DT implementations, while recent membrane-specific work highlights the potential for ML-enabled fouling prediction to support adaptive control, cleaning, and chemical-dosing decisions [36,65]. This layer also holds the human-in-the-loop governance that decides whether actions execute automatically or need operator approval. The degree of automated bidirectional interaction here is what separates a DS from a DT, which is consistent with the broader DT literature’s emphasis on continuous feedback between physical and virtual entities [24].

The proposed five-layer architecture synthesizes established DT components while adapting them specifically to membrane processes. Its contribution lies in integrating ML-based state estimation, XAI, fouling forecasting, prescriptive control, and design–operate–maintain feedback into a unified framework. Thus, the novelty is primarily in the membrane-specific integration, functional layering, and progression toward increasingly autonomous decision-making, rather than in individual DT components themselves.

### 4.2. Data Flow as a Closed Loop

**Figure 3:**
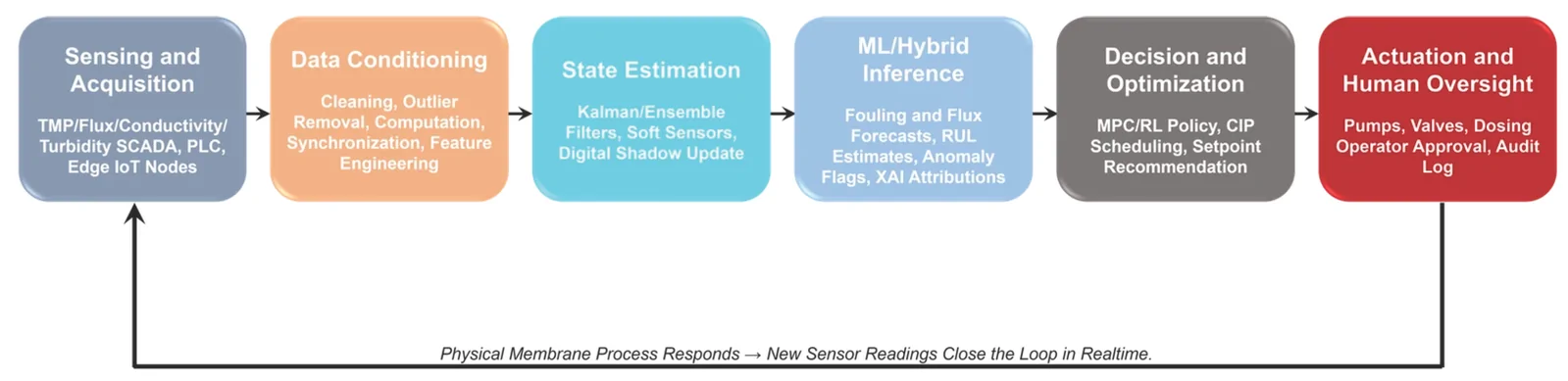
Closed-loop data pipeline from sensing to actuation in a membrane digital twin.

Intelligent membrane systems are often drawn as a one-way pipeline from sensing to decision. Figure 3 instead shows the closed loop of acquisition, conditioning, state estimation, inference, decision, and actuation: measurements update the virtual representation and DT outputs return to the physical system, whose response becomes the next cycle’s input [81]. This matters in membranes because feedwater quality, fouling state, and hydraulics change continuously. It also sharpens the DM/DS/DT distinction: a system whose outputs need human interpretation and manual action is a sophisticated DS, however advanced its modeling, while automated feedback through actuators or controllers is the defining pathway to a DT [82]. Recent wastewater DT frameworks implement exactly this chain from sensing and modeling through predictive control to plant controllers [83].

### 4.3. Interoperability and Standards

A persistent challenge in scaling intelligent membrane architectures is the limited availability of membrane- and water sector-specific standards for data models, semantic interoperability, DT communication, and validation. Although the International Organization for Standardization (ISO) 23247 provides a general DT framework for manufacturing, including principles, reference architecture, and information exchange requirements, it is not specific to membrane or water treatment processes. ISO 23247-2:2021 defines a reference architecture, while ISO 23247-4:2021 specifies technical requirements for information exchange between entities within the architecture [84,85,86]. Recent water treatment research has therefore explored modular and microservices-based DT architectures, allowing functional components such as process models, sensor interfaces, analytics, and optimization modules to be developed and updated independently. For example, Rodríguez-Alonso et al. [87] developed a microservices-based DT platform for wastewater treatment designed for edge computing implementation. More broadly, wastewater DT reviews identify integration of heterogeneous sensors, mechanistic and data-driven models, IoT infrastructure, edge/cloud computing, and control systems as important architectural requirements [24]. The standardization landscape has also recently progressed. ISO 23247-5:2026 establishes principles for digital threads that connect, manage, and maintain digital twin information across lifecycle stages, while ISO 23247-6:2026 introduces approaches for composing multiple DTs through integrated, unified, or federated configurations to improve interoperability and scalability [88,89]. Nevertheless, these standards remain manufacturing-oriented and their applicability to heterogeneous membrane processes, water sector data semantics, model validation, and cybersecurity requires systematic evaluation. Consequently, the absence of mature membrane-specific interoperability and governance frameworks remains a first-order barrier to large-scale DT deployment.

## 5. The ML Methods Landscape

### 5.1. Perception and State Estimation

Before forecasting or control, a DT must estimate the current state of the membrane system from noisy, incomplete, or indirect measurements, which is a state- and parameter-estimation problem rather than a conventional prediction task. Kalman filter variants, including the Ensemble Kalman Filter (EnKF), recursively assimilate measurements while accounting for process and measurement uncertainty [90]. Thosar et al. [91] combined EnKF with an adaptive PINN, so that states and parameters update online; the DT held predictive performance under parameter change and model–plant mismatch with far lower online computation than a physics-based EnKF. This adaptability suits membranes, where feed composition, hydraulic loading, and fouling vary widely. Soft sensors infer hard-to-measure states from routine measurements. Online fouling-resistance estimation by recursive methods has enabled monitoring of fouling development and prediction of cleaning needs [36,92], and live digital shadow implementations have combined mechanistic filtration models with Kalman filtering to infer membrane resistance [93]. Latent variables such as membrane resistance, fouling state, and effective permeability can thus be reconstructed continuously from SCADA data, forming the foundation for forecasting, diagnosis, and closed-loop decisions.

### 5.2. Predictive Forecasting

Forecasting fouling, TMP, permeability, and energy demand is the most studied ML function in membranes, and the choice between classical and deep approaches depends on dataset size, temporal structure, and quality. A comparison of multiple linear regression, ANN, and SVR for TMP at a seawater desalination plant used 195 daily observations; synthetic augmentation enlarged the training set, but only 39 independent real observations were kept for testing, which is a warning against over-interpreting small or poorly time-resolved datasets [94]. Long records allow more: Roehl et al. [29] trained ANNs on six years of full-scale RO data (59 hydraulic and water quality variables, 190 inter-cleaning runs), separating initial particle deposition from later biofilm and scale growth. Different predictors dominated each phase: chlorine, electrical conductance, and total dissolved solids early; turbidity, nitrate, and organic nitrogen later.

Deep sequence models are increasingly being applied where temporal dependencies are important. A recent MBR study using an LSTM encoder–decoder architecture reported MAPE below 6.45% and R^2^ above 0.87 for membrane-fouling forecasting, demonstrating the potential for proactive cleaning decisions rather than reactive responses to performance deterioration [26]. Similarly, recent SWRO research combined non-autoregressive multiresolution imputation (NAOMI) with deep learning models to address sensor gaps before jointly predicting TMP, energy consumption, and permeate flow [95]. This emerging emphasis on joint forecasting, missing data treatment, and explainability is particularly relevant to real plants, where correlated performance indicators and imperfect sensor streams are unavoidable.

### 5.3. Diagnosis and Attribution

A forecast does not explain why fouling risk is rising. This diagnostic role is increasingly filled by XAI, chiefly SHAP and, less often, LIME. Liang et al. [47] used SHAP to identify dominant operational and biological fouling drivers [45], and explainable transfer learning has used SHAP with leave-one-feature-out analysis to separate transferable fouling signals from plant-specific drivers [96]. Attribution is diagnostic evidence, not proof of causality: SHAP values are model-dependent, and a highly ranked feature may merely correlate with the true driver, so results should be checked against known fouling mechanisms and, where possible, independent physicochemical measurements [96]. XAI is the interpretive bridge between Layer 4 (reasoning) and Layer 5 (actuation): physically plausible explanations make predictions suitable for operator review, auditing, and higher automation, and DT reviews identify interpretability, uncertainty, and auditability as prerequisites for trustworthy AI-enabled operation [97].

### 5.4. Prescriptive Decision-Making

Reinforcement learning (RL), genetic algorithms (GAs), Bayesian optimization (BO), and model-predictive control (MPC) informed by learned or hybrid models take analytics from forecasting to action, balancing fouling, energy consumption, and treatment performance when setting operating conditions, cleaning strategies, and dosing. GAs and other optimizers have tuned membrane operating parameters, and RL and BO are emerging for adaptive membrane regeneration and optimization [11,98]. This capability is less mature than prediction, particularly for validated plant-scale control: RL reviews in wastewater cite implementation, data availability, integration with process models, and operator trust as persistent barriers [99]. Mechanism-aligned ML, fallback control, and edge-based DTs point to safer deployment [34]. Prescriptive intelligence is therefore a major near-term opportunity, but progression from simulation to safe, validated closed-loop operation is essential.

Table 3 maps representative ML methods to their functions in membrane systems, showing where evidence is strong and where it is limited.

## 6. DT–ML Integration for Real-Time Process Optimization

### 6.1. From Open-Loop Forecasting to Closed-Loop Control

Forecasting fouling or flux decline, even with high accuracy, does not by itself determine the appropriate operating response, such as the pressure setpoint, antiscalant dosage, or timing of a CIP cycle. Real-time process optimization extends predictive intelligence by coupling the DT to an optimization or control layer that evaluates and, in advanced implementations, automatically executes corrective actions [11,31]. The DT continuously assimilates process measurements to update the estimated membrane state and generate short-horizon performance forecasts. An optimization layer, typically based on MPC or RL, then evaluates alternative actions against defined performance objectives and operational constraints [34]. The selected action may be implemented automatically through the plant control system or submitted to an operator for approval. The resulting process response is subsequently measured and fed back into the DT, allowing its state estimate and subsequent decisions to be updated continuously. Such feedback-enabled optimization is a defining pathway toward autonomous membrane operation, although validated plant-scale applications remain less mature than predictive fouling models [65].

### 6.2. Model Predictive Control in Membrane Operation

MPC has a long history in RO, classically minimizing specific energy consumption while holding permeate quality and recovery within bounds. The value of DT coupling lies not in the control algorithm but in the fidelity and adaptivity of the model: conventional MPC relies on a periodically recalibrated model, whereas a DT-coupled controller continuously updates estimates of permeability, fouling state, and feedwater conditions, accommodating drift without re-identification. ML models within MPC for closed-circuit RO have shown the feasibility of learned dynamic models for real-time energy minimization [101]. Gao et al. [102] field-tested a two-level energy-optimal controller for two-stage RO that used real-time feed salinity and membrane permeability to set recovery and pressure while maintaining productivity; earlier work showed self-adaptive energy-optimal control under fluctuating salinity [103]. Savings of up to 17% were reported for a two-stage SWRO plant [104], but such figures depend on the baseline and constraints and should not be generalized.

### 6.3. Reinforcement Learning for Membrane Process Control

Reinforcement learning (RL) lets an agent learn an operating policy through repeated interaction with a simulated or historical environment, which is attractive for nonlinear, constrained RO dynamics under changing feed and fouling. Deep Deterministic Policy Gradient (DDPG) and Deep Q-Network (DQN) agents have coordinated permeate flow, setpoints, energy use, and water demand requirements [105]; DDPG has manipulated TMP directly [106]; and multi-agent RL has extended the approach to two-stage RO, with agents coordinating plant-level objectives [107]. Multi-objective RL optimizes energy together with recovery, permeate quality, or fouling. A closed-circuit RO study pairing a plant-calibrated dynamic simulator with RL reported better specific energy consumption and recovery than rule-based control, with SHAP analysis for interpretability [108]; thermodynamics-guided surrogate-assisted RL for hybrid MSF–RO shows how physics-informed surrogates reduce exploration cost [107]. The main limitation is the simulation-to-reality gap. Many membrane RL studies are validated only against mathematical or dynamic models [105,106], so sensor noise, unmodeled disturbances, membrane aging, and changing feedwater can erode policy performance. Moon et al. [108] argue that calibrated simulators are needed because trial-and-error learning on operating plants is constrained by safety. Physical-plant deployment remains far less mature than simulation.

### 6.4. Dosing and Chemical Optimization

Adaptive dosing of antiscalants, coagulants, and cleaning agents in response to membrane condition and predicted fouling or scaling risk is a narrower but practical application. Ridwan et al. [109] built a neural network coagulant-dose optimizer for an industrial SWRO plant, reporting 95% accuracy for silt density index prediction and an 11.7% cut in annual coagulant use. Real-time optimization is also possible without ML. A smart antiscalant algorithm on two pilot RO units cut antiscalant use by 85–90% relative to supplier-recommended doses while controlling calcium carbonate scaling, and because under- and overdosing both harm performance, dosing must stay within scaling, fouling, and quality limits [110]. Dosing is therefore a practical intermediate step to autonomy, in which predictions become bounded actions while higher-level decisions stay under operator oversight.

## 7. DT–ML Integration for Fouling Management

### 7.1. Fouling Mechanisms as Digital Twin State Variables

**Figure 4:**
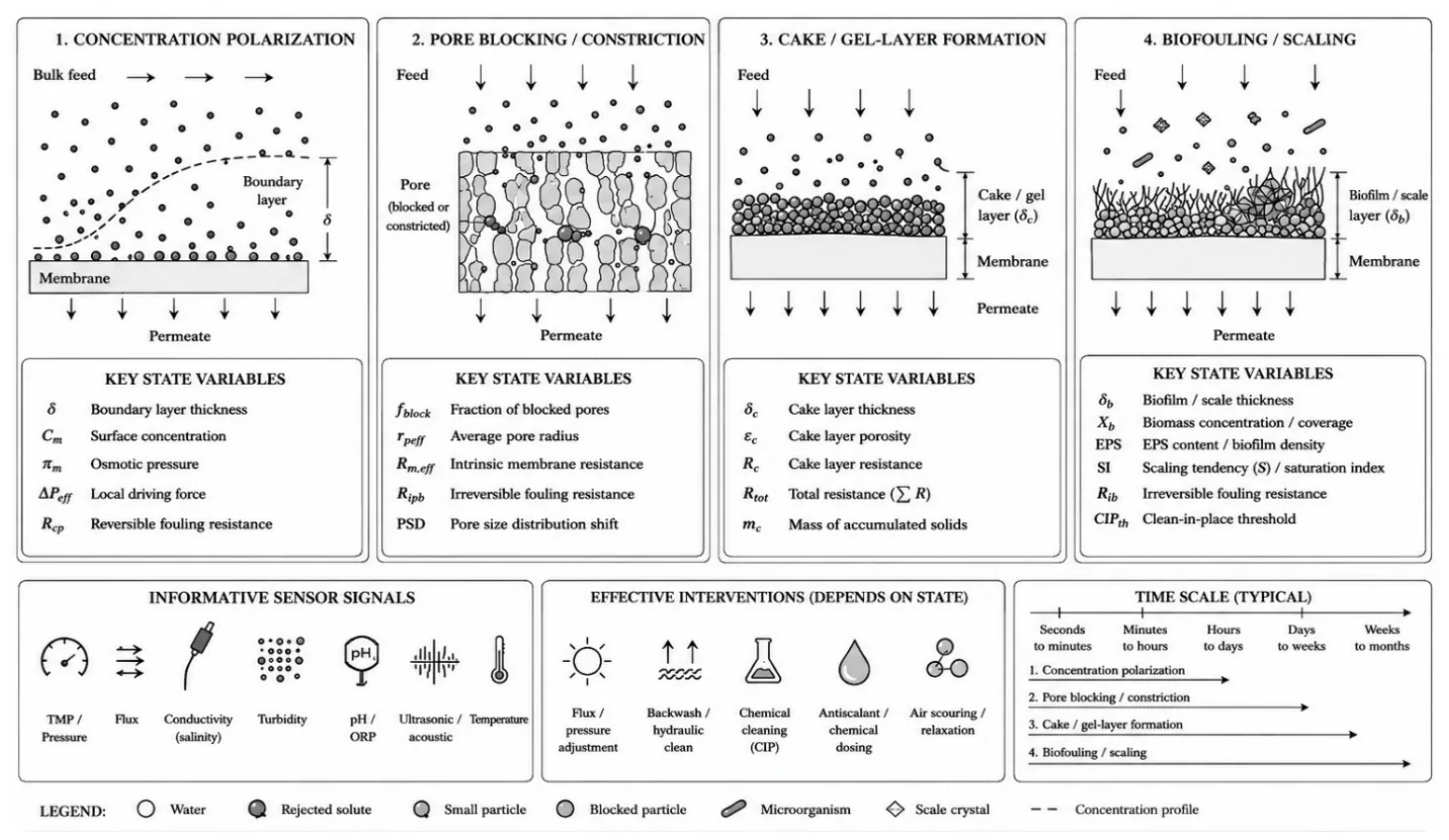
Principal membrane fouling mechanisms represented in digital twin state variables.

A DT that manages fouling must represent, explicitly or implicitly, how fouling occurs, since mechanisms determine which signals are informative and which interventions work. Fouling is the accumulation, adsorption, or deposition of suspended, organic, inorganic, and biological material on or in the membrane, causing flux decline and rising resistance or pressure [29]. As shown in Figure 4, four mechanisms recur: concentration polarization (solute build-up at the boundary layer, raising osmotic pressure and cutting driving force); pore blocking or constriction; cake or gel-layer formation, adding hydraulic resistance; and biofouling or scaling, which develops over longer time scales and resists hydraulic cleaning, often requiring chemical treatment. They are not mutually exclusive: polarization promotes deposition and precipitation, and deposits alter local mass transfer. Fouling is therefore a dynamic composite state, not a single measurable variable. Resistance-in-series formulations represent multiple resistance contributions, while hybrid and ML models infer the evolving state from TMP, flux, and water quality changes. A DT must link measured performance to plausible physical states rather than merely detect statistical deterioration, and full-scale evidence shows the value of distinguishing temporally evolving regimes [29].

### 7.2. An ML-Enabled Fouling Management Workflow

**Figure 5:**
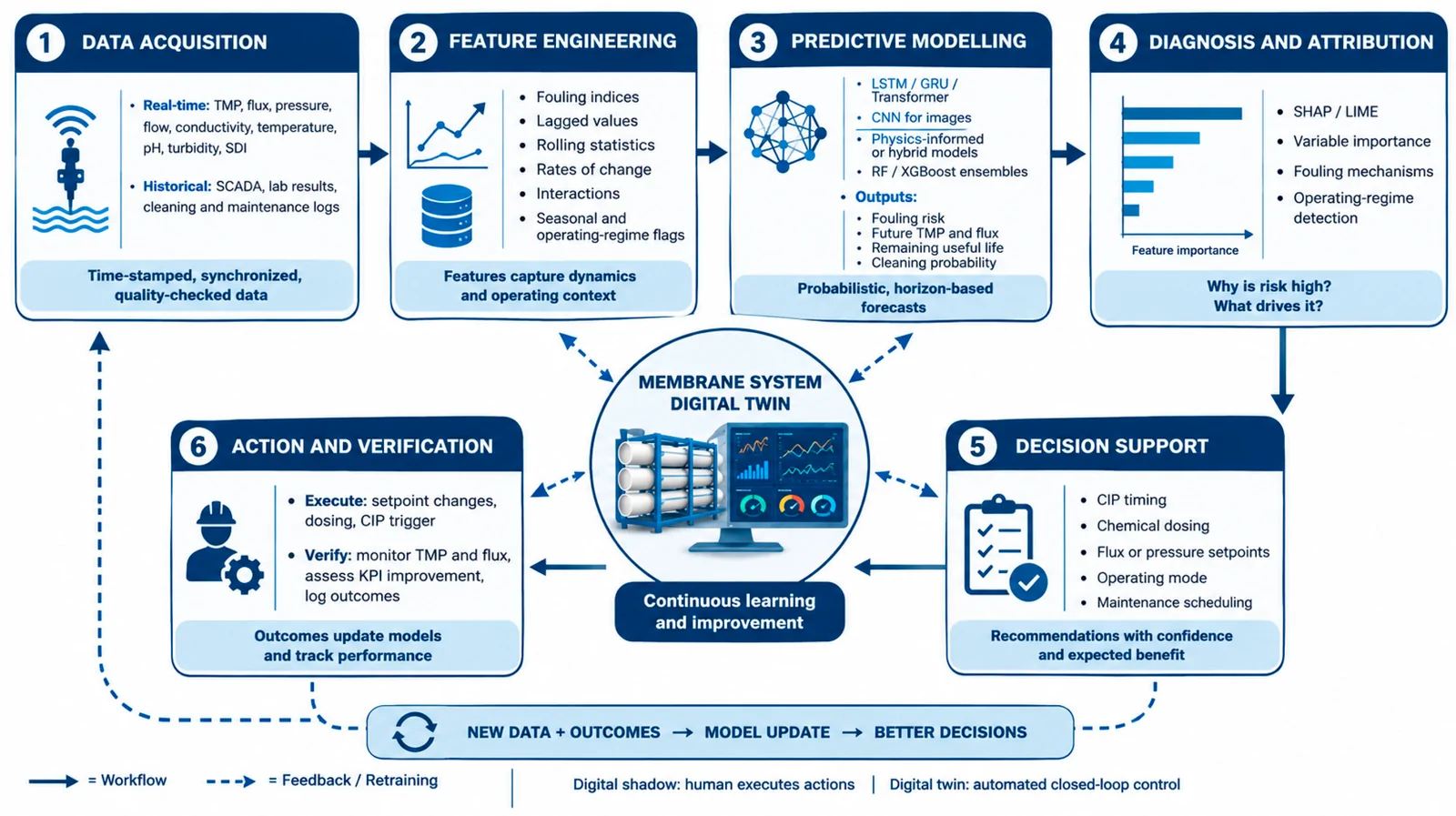
ML-enabled fouling management workflow within a digital twin.

Figure 5 illustrates the six-stage fouling management workflow implied by the DT architecture in Section 4. It begins with historical and real-time data acquisition, followed by feature engineering to derive fouling indices and lagged TMP/flux variables and predictive modeling using the architectures reviewed in Section 5 and Section 8. The resulting predictions are subjected to SHAP/LIME-based diagnosis and attribution before being converted through decision support into cleaning, dosing, or flux/pressure recommendations. Finally, action and verification close the loop, with interventions performed by operators or automatically by advanced DTs, and the resulting outcomes are fed back to improve the models.

### 7.3. Early Warning and Remaining Useful Life

An important operational output of this workflow is the estimation of remaining useful life (RUL) before a membrane element or cleaning cycle reaches a predefined performance limit. Such limits may include a specified flux decline, maximum tolerable transmembrane pressure, or minimum acceptable permeate quality. Unlike conventional point forecasting, RUL reframes fouling prediction as an estimate of the remaining operating time before intervention, closely linking the DT to maintenance scheduling and spare-parts management [47,80]. This reflects the broader evolution of AI-based fouling research from predicting membrane deterioration toward generating predictions that can directly support operational and economic decisions.

### 7.4. Autopsy-Informed Model Validation

Membrane autopsy is an important but under-used source of ground truth for ML fouling diagnosis. Combining SEM/EDS, FTIR, elemental analysis, and microbiological methods, it distinguishes organic/biological fouling, inorganic scaling, and colloidal deposition, giving an independent test of data-driven mechanism attribution [111,112]. Full-scale RO autopsies have found substantial organic and microbial-derived fouling with Fe-, Ca-, and Si-associated deposits, Ca, Mg, Al, Fe, and Si as key scaling elements, and bio-derived organic matter as a major foulant fraction [113,114]; earlier work showed heterogeneous, sequential organic–inorganic deposition and aluminum silicate scaling, so multiple mechanisms coexist and evolve [115]. Yet such evidence is rarely built into ML diagnosis workflows, leaving a validation gap between inferred mechanisms and actual deposits. Periodic autopsy could provide an independent validation layer for DTs.

### 7.5. From Reactive to Proactive Cleaning-in-Place (CIP) Scheduling

Where forecasting is coupled to a decision layer, CIP scheduling is among the most developed applications of predictive and RL methods. A full-scale industrial RO study combined attention-based long-sequence forecasting (R^2^ = 0.82) with RL to evaluate CIP pressure thresholds against cleaning cost and membrane life, cutting operating cost by 16.13% and extending operating time by 139.53% relative to the prior strategy [116]. For UF, Park et al. [117] built a deep-RL framework controlling pressure, cleaning time, and chemical concentration simultaneously, reducing specific energy consumption by about 20.9%. MBR cleaning decision models predict flux and recovery while weighing reliability and economics [118]. These results show a shift from calendar- or threshold-based to condition-based, economically informed cleaning. Benefits remain strongly dependent on baseline strategy, cleaning costs, membrane condition, and constraints, so CIP optimization is an intermediate step, not evidence that plant-wide autonomous control exists; reviews likewise call for further plant-scale validation [34].

Table 4 summarizes representative DT and hybrid-model case studies, classifying each by process, scale, and DM–DS–DT class for membrane fouling management.

## 8. Multi-Scale and Physics-Informed Hybrid Modeling

### 8.1. Why Pure Data-Driven Models Are Not Enough

Purely data-driven models can be accurate within their training domain but have two limitations for membrane DTs. First, generalization under distribution shift: models trained on a narrow range of feedwater quality, membrane condition, or operating conditions may fail under unusual salinity, membrane aging, or different setpoints, and reviews identify limited transferability and data scarcity as persistent barriers [34,120]. Second, black-box models do not encode the physics of transport and fouling, which makes their predictions hard to interpret and constrain in autonomous decisions. Physics-informed and hybrid approaches address both by building governing equations, mechanistic relationships, or physically meaningful descriptors into the learning framework. Hybridizing neural networks with the four Hermia fouling equations improved flux decline prediction, even with substantially reduced datasets while estimating the relative contribution of each fouling mechanism [35], and physics-guided ML using roughness-corrected interfacial descriptors outperformed purely data-driven models for flux decline and recovery [121]. Hybrid modeling is thus not a contest between mechanistic and data-driven approaches but a way to combine physical consistency, adaptability, and computational efficiency in one DT.

### 8.2. Physics-Informed Neural Networks (PINNs)

**Figure 6:**
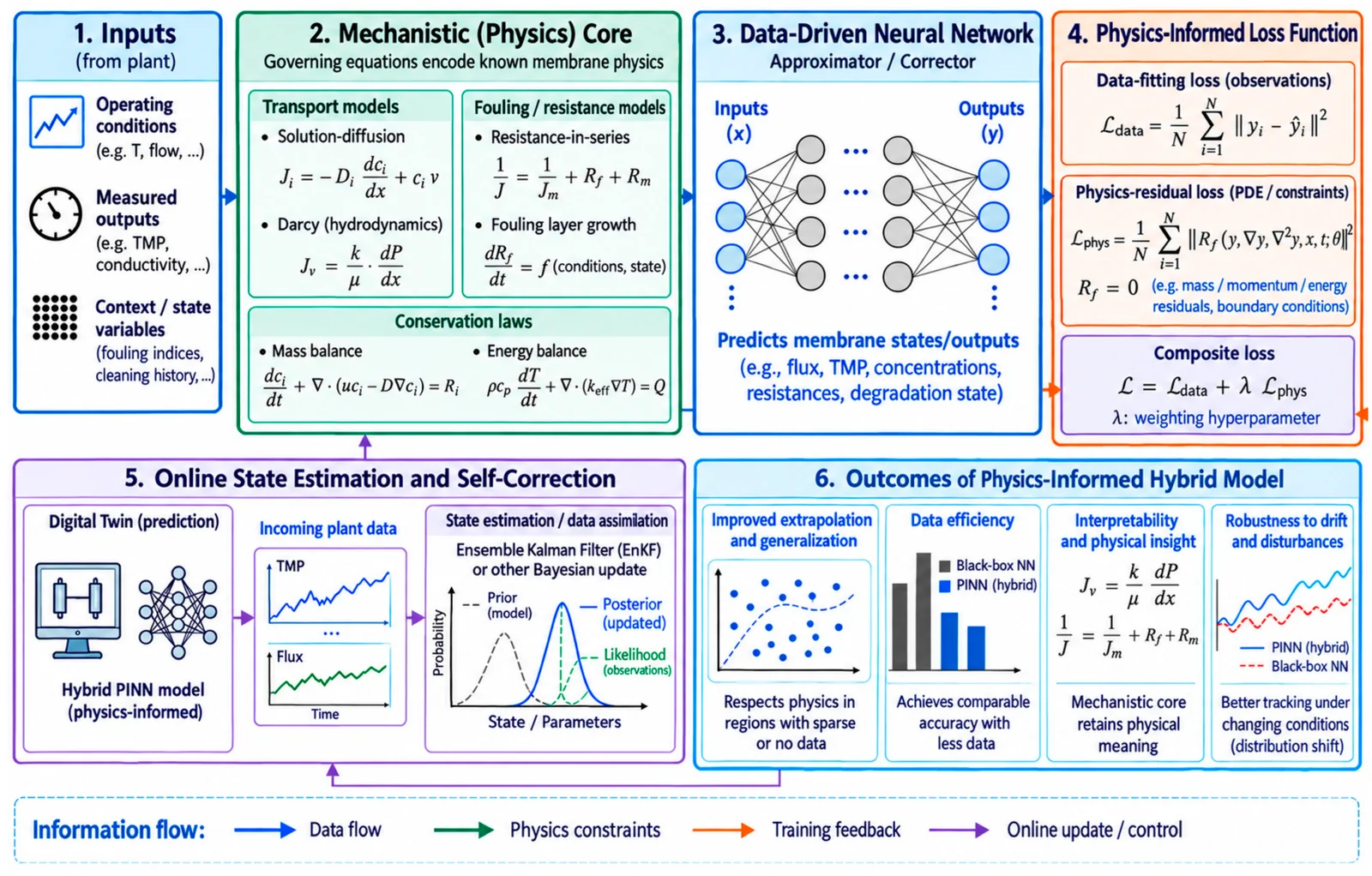
Physics-informed/hybrid modeling schematic for membrane digital twins.

A PINN adds governing relationships (mass, momentum, and energy balances) to the training objective, penalizing physical violations as well as prediction error, so that it learns from data while remaining consistent with process physics. PINNs have been applied to RO membrane degradation, using mass- and momentum-transport relationships to learn physically meaningful parameters and diagnose degradation mechanisms [122], and to fouling prediction by embedding Hermia laws, allowing identification of transitions between fouling mechanisms [35,123]. Hybrid architecture (illustrated in Figure 6) couples a mechanistic core (solution–diffusion, Darcy-based transport, resistance-in-series, and mass/energy balances) with a neural network through a composite objective containing data and physics-residual terms. Their relative weighting sets the balance between empirical fit and physical consistency. Adaptive PINN–EnKF DTs add online assimilation, updating states and parameters to cope with model–plant mismatch and drift [91]. PINNs thus offer data-efficient, physically constrained, and adaptive DT models, though extrapolation must be validated outside the training domain rather than assumed.

### 8.3. Grey-Box and Residual Modeling

A lighter hybrid strategy keeps an established mechanistic model as the primary predictor and trains an ML model on the systematic discrepancy between prediction and observation. The physical structure is preserved while the data-driven part captures hard-to-formulate effects such as unmodeled fouling, aging, and site-specific variability [124]. Hybrid membrane models have long combined mechanistic filtration relations with neural networks [125], and recent work used residual learning to correct the smooth-surface assumption in extended Derjaguin–Landau–Verwey–Overbeek (XDLVO) theory before passing the descriptors to an ML model for flux decline and recovery. The physics-guided model beat purely data-driven alternatives but degraded on previously unseen foulants [121]. For DTs, the practical advantage is that the mechanistic core stays largely intact while the residual model is periodically retrained as plant data accrue, which is a low-complexity route to adaptive, physics-aware twins wherever transport or resistance-in-series models already exist.

### 8.4. Multi-Scale Modeling Across the Membrane Lifecycle

**Figure 7:**
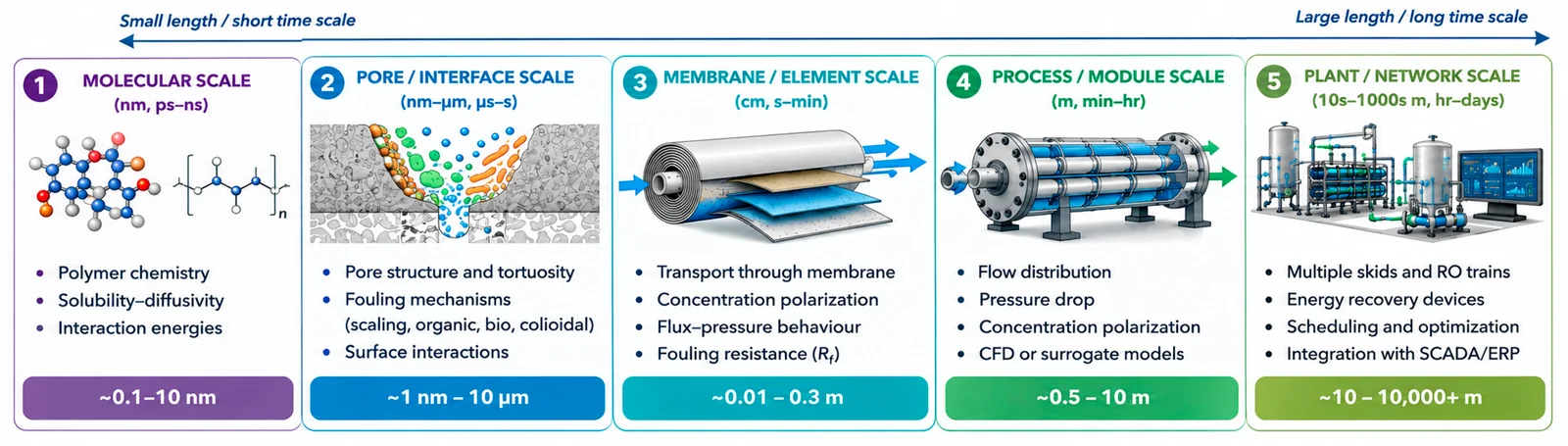
Multi-scale modeling hierarchy underpinning membrane DTs: from molecular chemistry through to plant-wide network operation.

Multi-scale modeling addresses at what structural scale behavior is represented. A membrane DT can be viewed as five linked levels (Figure 7) [36,126], with ML connecting information between them:

- Molecular: Molecular dynamics and solubility–diffusivity descriptors inform material selection and transport property prediction (linking to Section 9).
- Pore/interface: Adsorption, pore blocking, concentration polarization, and foulant–surface interactions supply the mechanisms behind the fouling states of Section 7.1; multiscale simulation connects molecular interactions to mesoscopic behavior [127].
- Membrane/element: Mechanisms become measurable permeability, flux, pressure drop, and rejection for spiral-wound or hollow-fiber elements.
- Process/module: CFD resolves flow distribution, spacer mixing, and polarization in channels, and CFD-trained ML surrogates reproduce pressure loss and mass transfer coefficients at far lower cost [128].
- Plant/network: Multiple trains, energy-recovery devices, utilities, controls, and scheduling form a plant-wide DT linked to SCADA and information systems [126].

The central challenge is scale bridging: information from expensive high-fidelity models must reach higher scales without making real-time operation impractical. ML surrogates and reduced-order models learn mappings from high-fidelity simulations and give rapid online predictions, a combination reviews identify as promising for real-time multi-scale DTs [129,130]. Such surrogates depend on the coverage of their training domain, so extrapolation to unfamiliar geometries, feed conditions, or degradation states needs explicit validation and uncertainty monitoring.

## 9. Towards Autonomous Design: Self-Driving Laboratories and Inverse Design for Membranes

### 9.1. Reframing Materials Discovery as a Digital Twin Problem

Materials discovery has developed largely independently of the DT literature, yet both fit a closed-loop cyber-physical framework: a system is characterized, represented computationally, evaluated against objectives, and acted upon to generate new information. Experiments give candidate properties, models capture structure–property relationships, an optimizer selects candidates, and synthesis and characterization generate data that update the model. This design–model–experiment–learn cycle parallels the sensing–modeling–decision–actuation–feedback architecture of an operational DT [17,131], so an autonomous materials platform can be seen as a DT of the design process itself, with a virtual model predicting permeability, selectivity, stability, or fouling resistance and the laboratory supplying validation and new observations. This is consistent with reviews positioning ML-assisted inverse design as moving from performance specifications to candidate structures instead of trial-and-error synthesis [70]. As automation grows, human involvement shifts from individual experiments to supervising objectives, constraints, and safety. Discovery systems should not automatically be called mature DTs; however, automated synthesis, characterization, data integration, and feedback must be demonstrated first, and the SDL literature stresses that current systems span different levels of automation and human involvement [132].

### 9.2. Self-Driving Laboratories

**Figure 8:**
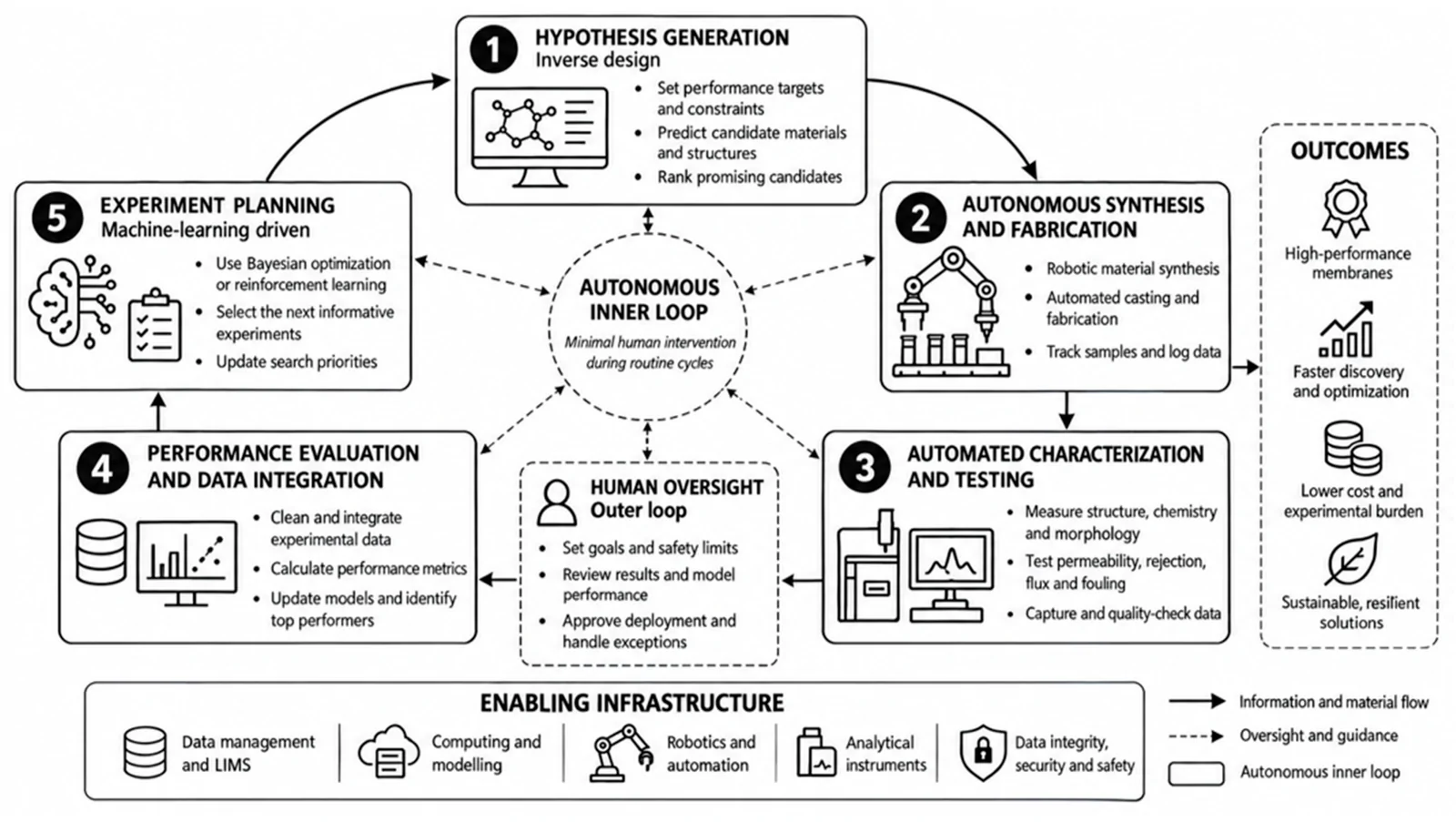
Closed-loop self-driving laboratory for membrane materials discovery.

Self-driving laboratories (SDLs) supply the experimental infrastructure: automated synthesis or fabrication, robotic handling, characterization, data management, and algorithmic experiment selection in an iterative workflow (Figure 8) [133]. BO, active learning, and, increasingly, RL select experiments that maximize information gain or improve target properties, and SDL development has progressed from automating single tasks to integrated, multi-step, multi-objective workflows [131,132]. For membranes, the chain would run from formulation through fabrication, characterization, and performance measurement to model updating and next-candidate selection, with forward and inverse design proposing chemistries, compositions, or fabrication conditions and automated characterization returning permeability, selectivity, flux, rejection, and stability [17,70].

Fully autonomous membrane SDLs are less developed than SDLs in broader chemistry and materials science, which reflects current evidence rather than an absolute absence of automation. Membrane fabrication adds challenges: reproducible control of phase inversion, interfacial polymerization, crosslinking, solvent exchange and post-treatment, and performance evaluation that can take far longer than a single characterization [134]. The realistic near-term route is the human-supervised SDL, with automated experiment selection and characterization but human approval of synthesis conditions and safety-critical decisions, in line with the SDL literature on human-in-the-loop operation [132].

### 9.3. Forward and Inverse Modeling of Membrane Materials

**Figure 9:**
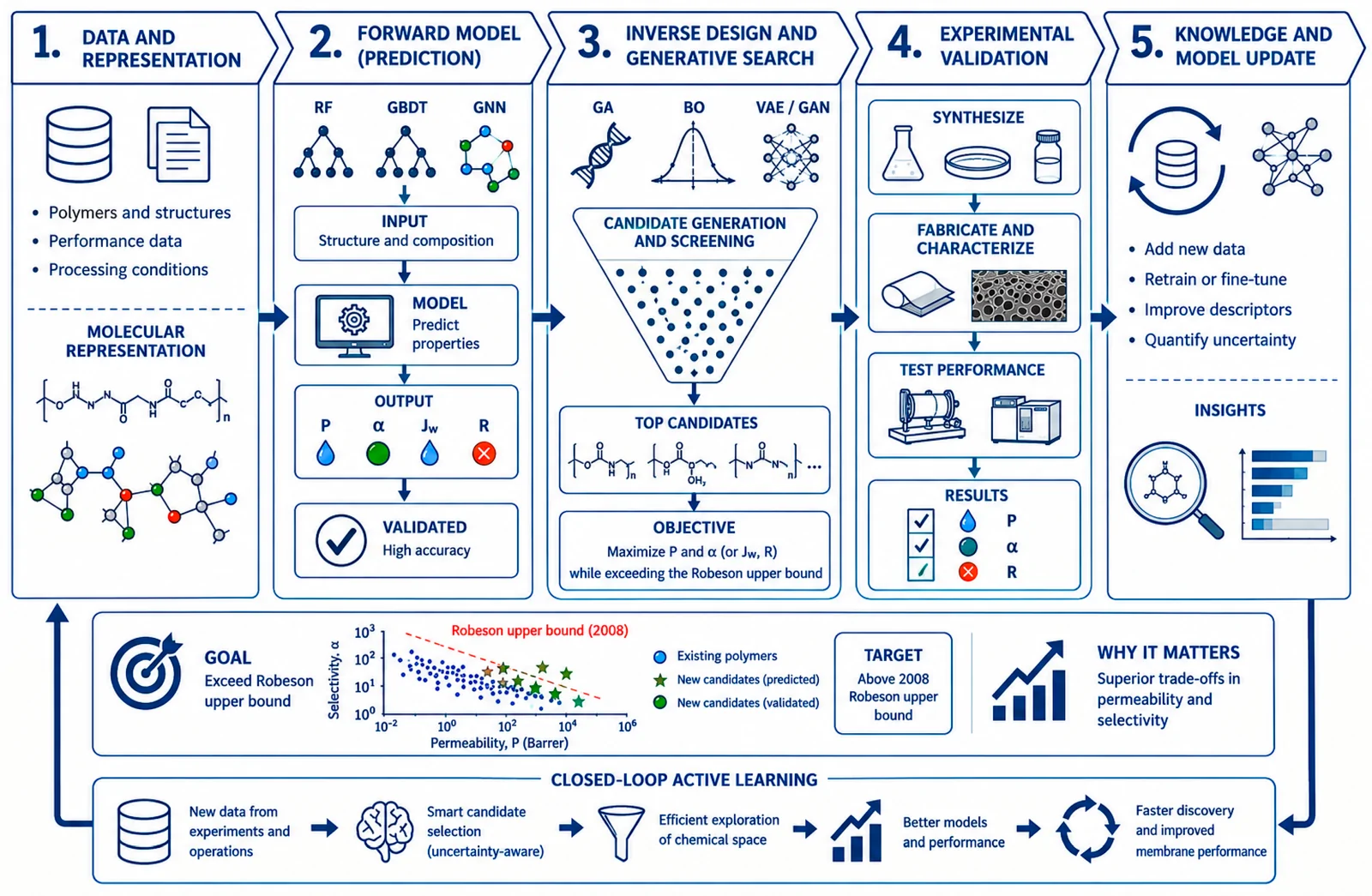
Conceptual illustration of a forward model/inverse design workflow for membrane material discovery.

Forward models predict membrane properties from a structure or composition; inverse design searches chemical space for materials meeting performance targets. Random forests, gradient boosting, and graph neural networks (GNNs) predict gas permeability and selectivity from polymer fingerprints, descriptors, and graphs, enabling computational screening before synthesis; graph models combining experimental and simulation data predict diffusivity, solubility, and permeability across polymer systems [135]. Inverse design couples a validated forward predictor to an optimizer or generative algorithm that proposes candidates meeting permeability–selectivity targets (Figure 9). ML with Bayesian molecular design has identified polymers predicted to exceed the CO_2_/CH_4_ and CO_2_/N_2_ Robeson bounds, with molecular dynamics for added validation [18], and ML-guided GAs generated over 16,000 candidate polymers, with some predicted to exceed both CO_2_/N_2_ and CO_2_/O_2_ bounds [41]. The aim is increasingly experimentally validated discovery: ML screening found candidates above the 2008 CO_2_/CH_4_ bound, two of which were synthesized and confirmed to exceed it, and explainable graph ML combining prediction, simulation, and synthesis has found polymers beyond empirical limits for several gas pairs [136]. Inverse design thus offers a direct pathway from desired performance to structure to experimental validation, paralleling the closed-loop autonomy framework for DTs.

### 9.4. Sustainability-Aware and Multi-Objective Design

Design objectives are widening beyond permeability and selectivity to environmental, functional, and manufacturing attributes. Workflows optimizing a single metric can yield candidates that are hard to make, operate, or dispose of: biodegradability, toxicity, embodied impact, mechanical stability, and processability are often ignored. One study incorporated biodegradability alongside CO_2_ permeability and selectivity, screening 3219 candidates and evaluating about 500 that exceeded Robeson bounds for biodegradability [19]. It should not be described as full multi-objective optimization because independently trained permeability/selectivity and biodegradability models were combined by sequential screening rather than a Pareto or Bayesian multi-objective optimizer [19]. Future autonomous design could extend the objective to separation performance, biodegradability, embodied carbon, robustness, thermal stability, processability and cost, using Pareto-front or multi-objective BO to manage trade-offs.

### 9.5. Closing the Loop Between Operation and Design

A major opportunity identified here is to connect operational DTs with autonomous discovery platforms. Operational DTs assimilate performance, fouling evolution, operating conditions, energy use, and maintenance data, giving application-level evidence about what determines membrane lifetime and efficiency [120]; this can specify the permeability, chemical stability, fouling resistance, and durability that new materials must meet, while inverse design frameworks generate candidates from targets and narrow the experimental search [19]. An integrated operation–design–validation loop would have plant data define requirements, inverse design generate structures, automated laboratories synthesize and characterize them, and DTs predict their behavior under realistic conditions [137]. This remains a research opportunity, not industrial practice: reviews still cite data scarcity, heterogeneous datasets, limited transferability, and the gap between computed and experimentally validated materials [17]. Moreover, Ghasemzadeh et al. [36] note that fully autonomous closed-loop operation remains largely unrealized at the industrial membrane scale. Interoperable data structures, shared performance descriptors, and bidirectional links between operational DTs and discovery systems would be the foundation for self-improving platforms in which operational experience informs the next generation of materials.

## 10. A Proposed Autonomy Framework for Intelligent Membrane Systems

**Figure 10:**
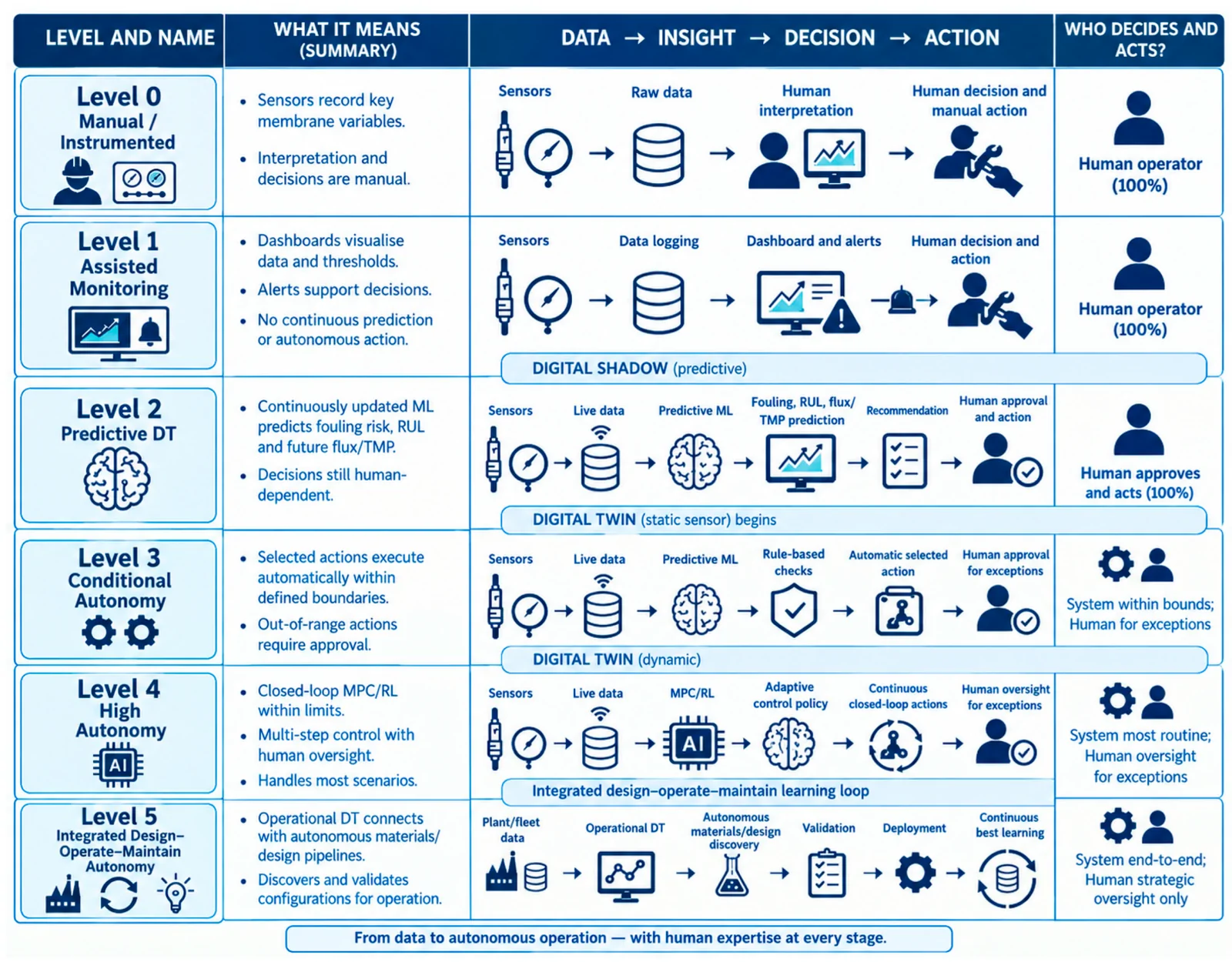
Proposed five-level autonomy framework for intelligent membrane systems from manual instrumentation through to full autonomy including design.

The automotive six-level taxonomy (SAE J3016) endures because it gives regulators, engineers, and the public a shared ordinal vocabulary for comparing very different systems by how much of the sensing-to-action loop runs without human mediation, within defined operating conditions. Membrane DTs have no equivalent scale, although a fouling-alarm dashboard, an RL dosing controller, and a self-driving materials laboratory might all be called “intelligent membrane systems”. This review proposes such a scale (Figure 10), spanning operational and design-stage autonomy so that the studies reviewed in Section 6 and Section 7, and 9 sit on one comparable axis.

Level 0 (manual/instrumented): At this level, sensors continuously or periodically record key membrane process variables, including TMP, flux, and conductivity, while interpretation and operational decisions are performed manually by plant personnel. The architecture contains no automated modeling or decision support layer linking sensor measurements to subsequent operational actions. This configuration remains common in conventional membrane facilities, particularly where digitalization and advanced process-control infrastructure are limited.

Level 1 (assisted monitoring): At this level, sensor data are automatically visualized through dashboards and evaluated against predefined thresholds to generate alerts when abnormal operating conditions occur. Although this introduces a degree of automated monitoring, the system does not continuously predict future process states or autonomously determine corrective actions. It can therefore be regarded as an early-stage digital shadow architecture, provided that a persistent digital representation and automated data connection to the physical process are present.

Level 2 (predictive DT): This level introduces continuously updated ML-based prediction, using live plant measurements to estimate fouling risk, RUL, and future flux or TMP behavior. The resulting predictions can support maintenance and operational decisions, but proposed interventions remain dependent on human review before execution. The literature reviewed suggests that many systems described as DTs operate within this predictive, human-supervised configuration, highlighting the importance of distinguishing predictive capability from autonomous closed-loop operation.

Level 3 (conditional autonomy): The system automatically executes selected operational actions within operator-defined safety and performance boundaries. For example, antiscalant dosing may be adjusted automatically in response to predicted fouling risk, while CIP scheduling or setpoint changes outside the predefined operating envelope remain subject to human approval. At this level, the architecture can meet the defining characteristics of a DT (in the strict Kritzinger sense) for the functions operating within the automated feedback loop, provided that the digital and physical systems remain continuously and bidirectionally coupled.

Level 4 (high autonomy): Here, closed-loop MPC or RL policies continuously translate predicted process states into adaptive operational multi-step or continuously updated control actions within a pre-validated operating envelope. Human intervention is primarily supervisory and is triggered by abnormal conditions, safety-critical events, policy violations, or states outside the validated envelope. Few of the case studies reviewed in Section 6 and Section 7 have been demonstrated at this level under field conditions, as opposed to simulation. The RL of validated field deployment is at this level.

Level 5 (integrated design–operate–maintain autonomy): This level expands autonomy beyond process operation to encompass materials and process design. The operational DT is linked to a self-driving-laboratory-style materials-and-design pipeline, allowing fleet-wide operating experience to inform autonomous materials discovery, while validated materials or operating configurations can be evaluated and incorporated into plant operation with minimal human mediation. Thus, Level 5 represents an integrated design–operate–maintain learning loop rather than simply greater control automation. No fully realized example was identified in the reviewed literature; it therefore represents the aspirational endpoint of the proposed roadmap.

Applying the framework to Table 3, the ANN fouling-phase model sits at Level 2 (it forecasts and diagnoses but does not act). The microservices wastewater DT platform can span Levels 1–3 depending on the loop considered, showing that autonomy is a property of individual control loops, not of a DT as a whole; a plant may be at Level 3 for dosing yet Level 1 for cleaning scheduling. The automated dosing case reaches Level 3. Deep-RL energy/fouling co-optimization reaches Level 4 in simulation but, in the great majority of reports, only Level 2 in field deployment, which this review regards as one of the most important and tractable near-term research gaps. No case reaches Level 5. The framework is an analytical and comparative device, not a certification standard or a checklist to be satisfied mechanically. Progression is not desirable everywhere: a small utility with limited technical capacity may rationally stay at Level 1 or 2. The appropriate level depends on the consequence of error, data quality and volume, and the regulatory and institutional context (Section 12).

## 11. Applications of the Proposed Framework Across Membrane Process Types

The autonomy framework proposed in Section 10 provides a common basis for assessing how far DT–ML integration has progressed across different membrane technologies. Rather than treating all reported DT applications as equivalent, this section uses the framework to distinguish between systems that merely monitor process conditions, those that generate continuously updated predictions, and those that actually close the sensing-to-action loop. This distinction is particularly important because technological maturity is not uniform across membrane processes: a process may have sophisticated ML-based fouling prediction while remaining at Level 2, whereas another may demonstrate automated control of a narrow operating variable at Level 3 without possessing broader plant-wide autonomy.

### 11.1. Reverse Osmosis (RO) and Seawater Desalination

RO, particularly SWRO, is the most mature area for operational DT–ML integration, reflecting high capital intensity, substantial energy use, and relatively extensive instrumentation, so that small gains in energy, productivity, dosing, or cleaning frequency carry large economic value. Examples of ML fouling prediction, automated dosing, and energy/fouling co-optimization concentrate here. Through the Section 10 lens, though, this maturity needs qualifying. Many systems remain at Levels 1–2, with automated monitoring or updated predictions but human authority over decisions. Automated dosing provides Level 3 evidence, since a defined action executes within preset bounds. Deep RL shows technical potential for Level 4, especially for joint energy–fouling optimization, but most implementations are simulation-based or heavily supervised in the field. The near-term opportunity is therefore not more accurate prediction but the move from Level 2 to validated Level 3–4 closed loops, requiring robust uncertainty estimation, explainable recommendations, safety constraints, fault detection, and long-duration field validation.

### 11.2. Nanofiltration (NF) and Ultrafiltration (UF)

NF and UF occupy an intermediate position. ML fouling prediction is comparatively well established given widespread use in drinking water, wastewater, and industrial separations, but evidence for continuous optimization and autonomous control is much thinner than for RO. Most applications sit at Levels 1–2, where data are visualized, abnormalities detected, or behavior predicted without automatic corrective action. Data-driven UF DTs point toward more integrated representations, yet evidence for Level 3 remains small. The challenge is less ML capability than limited integration of prediction with validated control, needing tighter coupling between fouling forecasts, hydraulic and chemical process models, and decision layers. Economic incentives for autonomous infrastructure may also be weaker, especially in smaller UF systems.

### 11.3. Microfiltration (MF)

MF is the least developed of the pressure-driven liquid processes, largely for economic and functional reasons: it is often a low-cost pretreatment step rather than the main value-generating separation, so the incentive for sophisticated DT infrastructure is lower. Most applications sit at Levels 0–1, dominated by instrumentation, monitoring, and threshold-based intervention. Low maturity does not imply low potential: because MF performance affects downstream RO or NF, integrating MF monitoring into a plant-level DT would make its state an upstream variable in downstream fouling prediction, which is a route from isolated monitoring to multi-process Level 2–3 autonomy.

### 11.4. Membrane Bioreactor (MBRs)

MBRs show a distinctive profile. ML fouling prediction is relatively well developed because fouling directly drives energy use, permeability, cleaning, and reliability, and explainable AI and DT work increasingly link predictions to interpretable decisions. Yet most of the MBR literature sits at Level 2, where models forecast but operators intervene; some systems approach Level 3 through automated control functions, but plant-wide autonomy is uncommon. The obstacle is partly mechanistic: MBRs combine membrane transport with complex biological, biochemical, and physicochemical interactions in mixed liquor, making robust hybrid modeling harder than for well-characterized RO. Progress to Levels 3–4 will require ML prediction combined with mechanistic representations of biological and fouling processes, uncertainty-aware decision support, and explainable control.

### 11.5. Membrane Distillation (MD)

MD remains comparatively immature across the autonomy framework. As an emerging technology that is less commercially established than RO and MBRs, it has accumulated fewer standardized long-term operating datasets and has comparatively limited large-scale deployment. Most MD applications therefore remain around Levels 0–2, with experimental monitoring and predictive modeling substantially more common than autonomous control. The principal constraint is consequently not necessarily the suitability of DT–ML approaches but the maturity of the underlying technology and its digital infrastructure. As MD moves toward larger-scale and more standardized operation, greater availability of operational data should create opportunities for progression toward predictive DTs and eventually conditional autonomous control.

### 11.6. Gas Separation and Pervaporation

These membranes show an almost inverted profile: materials design ML is far more mature than operational DT development, reflecting the field’s history in materials informatics, where ML has been widely applied to permeability, selectivity, and structure–property relationships. They are strong in the design domain but offer little evidence of Level 3–4 operational autonomy at the industrial scale. The key limitation is the weak connection between design pipelines and continuously operating membrane assets, which creates an opportunity for Level 5 integration: operational DTs could supply real-world information on degradation, selectivity, fouling, aging, and constraints; autonomous laboratories could use it to guide the search for better chemistries; and new materials could be evaluated in plant-level DTs before deployment.

### 11.7. Cross-Process Lessons

Technologies differ not only in how much DT–ML development exists but in where along the autonomy pathway it has occurred. RO and MBRs have strong operational capabilities, but most applications remain at Levels 1–2, with selected dosing and control approaching Level 3 and RL showing Level 4 capability mainly in simulation [14,16,26,29,101,102,103,104,105,106,107,108,109,110,138]. NF and UF are intermediate, with established prediction but fewer validated closed loops [36,39,100,117]. MF and MD are less digitally mature, reflecting limited datasets, deployment scale, and DT infrastructure [31,33,36,50,51,52,53,54,55,56,57,58,59,60]. Gas separation and pervaporation are the reverse: their strongest advances are in materials informatics, forward prediction, inverse design, and computational discovery, including polymers predicted or demonstrated to beat empirical limits [17,18,41,68,69,70,71,72,73,74,75,76], which are developed largely independently of plant-level DTs.

The result is a design–operation asymmetry: technologies with rich operational data have immature autonomous design pipelines, while those with sophisticated design capabilities have limited operational DT integration. Autonomy is therefore a property of a functional loop, not of a technology or plant. One RO or MBR facility may run at Level 3 for dosing, Level 2 for fouling prediction, and Level 1 for maintenance scheduling, while a gas separation membrane may show Level 4–5 characteristics in computational design yet low autonomy in plant operation. The framework thus exposes both maturity and where in the design–operate–maintain lifecycle it sits. Future progress should focus less on maximizing autonomy within each technology than on connecting disconnected capabilities: operational DTs generating requirements for fouling resistance, chemical stability, aging, energy use, and lifetime, and inverse design and SDL platforms turning them into candidate materials and fabrication conditions [17,19,70,131,137]. The goal is an integrated architecture in which discovery, operation, and maintenance inform one another, which bridges to the barriers and deployment roadmap.

## 12. Critical Analysis: Limitations, Barriers, and Open Questions

The literature reviewed in this study demonstrates substantial progress in applying ML, hybrid modeling, and DT concepts to membrane processes; however, the evidence also indicates that the transition from accurate prediction to reliable autonomy remains incomplete. The principal limitations are not associated with the absence of sophisticated algorithms alone. Rather, they arise from the interaction of data limitations, distribution shift, inadequate validation, interoperability constraints, cybersecurity risks, economic uncertainty, explainability requirements, regulatory concerns, and limited multidisciplinary expertise. These issues become progressively more consequential as membrane systems move from monitoring and prediction toward automated decision-making and, ultimately, autonomous materials design.

### 12.1. The Generalizability Problem

The most important methodological limitation is the restricted generalizability of reported models. Many studies use data from a single plant, membrane configuration, feedwater source, or campaign, and train and test on observations from the same operating environment. High R^2^ or low error then shows interpolation within one distribution, not robustness across plants, seasons, membrane ages, or feeds [29,31,34,59,94]. Fouling is particularly sensitive because interacting physicochemical and biological factors change in importance with feedwater and history: Roehl et al. [29] found different variables dominating different stages of fouling in a full-scale RO system, so the input–fouling relationship is neither static nor universally transferable. Han et al. [96] demonstrated explainable transfer learning for data-limited pilot-scale MBRs, transferring information between plants while identifying plant-specific drivers, but such studies are rare. Future work should move beyond random train/test splits to temporal, cross-season, cross-membrane, and cross-site validation, and external validation should be a minimum requirement for deployability claims. Where data cannot be shared, federated learning is promising, though it raises questions of model heterogeneity, communication cost, privacy, data quality, and security. The challenge is representative, traceable, and independently validated data, not just more data.

### 12.2. Data Scarcity, Quality, and Provenance

Many membrane studies use hundreds or thousands of observations, which is far fewer than vision or language applications, and compensate with feature engineering, synthetic data, and transfer learning [31,34,94]. More nominal observations do not mean more information: synthetic augmentation reproduces statistical patterns without new physical regimes, and dense sampling of one operating state may be less useful than a smaller designed dataset spanning temperature, salinity, recovery, flux, membrane age, and feed composition. Missing data, calibration drift, inconsistent sampling, and undocumented interventions further complicate historical SCADA data, and SWRO work shows that reconstruction of missing inputs is itself part of reliable prediction [95]. Studies should therefore report provenance, sensor reliability, missing data treatment, sampling frequency, operating domain coverage, and intervention history alongside performance metrics. Benchmark datasets with synchronized process, water quality, membrane condition, and maintenance information, together with common ontologies and metadata, are needed so that similar-looking variables do not hide different measurements, locations, or time scales [24,63,64,65].

### 12.3. Distribution Shift, Transferability, and Model Drift

Even a well-validated model may deteriorate after deployment because the membrane process evolves continuously. Membrane aging, seasonal changes in feedwater, changes in pretreatment, sensor replacement, altered operating strategies, and changes in cleaning chemistry can all cause the statistical distribution of the input variables to shift. This creates a fundamental distinction between a static predictive model and an adaptive DT. Hybrid modeling provides one possible response. Mechanistic models encode relationships that remain meaningful outside the precise training distribution, while ML components can learn plant-specific deviations [35,38,121]. Adaptive state estimation approaches, such as PINN–EnKF frameworks, provide another route by updating model states and parameters as new observations become available [91]. Nevertheless, online adaptation introduces its own risks. Continuous retraining can cause catastrophic forgetting, propagate erroneous sensor information into the model, or gradually move the model away from its validated operating envelope. Consequently, future DTs require explicit model lifecycle management, including drift detection, version control, retraining criteria, uncertainty thresholds, and rollback mechanisms.

### 12.4. Interoperability and Cybersecurity

A membrane DT may need to communicate with SCADA systems, PLCs, laboratory information systems, cloud platforms, edge devices, process simulators, and enterprise asset management systems. Without common data structures and communication protocols, each integration becomes a plant-specific engineering exercise, limiting scalability [24,65,87]. ISO 23247 provides a useful general framework for DT architecture and information exchange [84,85,86], while the more recent ISO 23247-5 and ISO 23247-6 developments address digital threads and composition of multiple DTs [88,89]. However, these standards were developed primarily for manufacturing and do not by themselves resolve membrane-specific requirements concerning water quality semantics, fouling state representation, model validation, or control system integration. Developing sector-specific ontologies and interoperable data models should therefore be considered a priority. Cybersecurity becomes increasingly important once DT outputs influence physical actuators. A predictive dashboard can tolerate a degree of communication failure; an automated dosing or pressure controller cannot. Compromised sensors, manipulated data streams, or unauthorized control commands could cause chemical overdosing, membrane damage, process instability, or non-compliant water quality. The literature reviewed here contains considerably more discussion of prediction and optimization than of cybersecurity-by-design. Intelligent membrane systems should therefore incorporate authentication, access control, encryption, anomaly detection, network segmentation, secure update procedures, and fail-safe operating modes from the beginning rather than treating cybersecurity as a post-deployment feature.

### 12.5. Explainability and Uncertainty

High predictive accuracy is insufficient when ML outputs influence safety-, quality-, or economically critical decisions. SHAP, LIME, permutation importance, and related XAI approaches provide useful mechanisms for identifying influential variables [45,46,47,96]. However, feature attribution should not be interpreted as proof of causality. A model may assign high importance to a variable because it is correlated with an unmeasured physical driver. Consequently, explanations should be evaluated against established fouling mechanisms, independent measurements, and mechanistic models. Uncertainty quantification should accompany explainability. An automated system should distinguish between a high-confidence prediction within its validated domain and an apparently precise prediction generated under unfamiliar conditions. Bayesian models, ensemble methods, conformal prediction, and probabilistic state estimation approaches provide potential mechanisms for quantifying predictive uncertainty. In an autonomous DT, uncertainty should ultimately influence the degree of automation: low-risk, high-confidence actions may be automated, whereas uncertain predictions should trigger operator review or revert to a conservative control strategy.

### 12.6. Techno-Economic Constraints and Regulatory Trust

Technical feasibility does not guarantee economically rational deployment. Developing a DT requires sensors, communications infrastructure, data storage, computational resources, software integration, cybersecurity, and personnel. These costs must be justified against measurable benefits, such as reduced energy consumption, chemical use, downtime, membrane replacement, and cleaning frequency. Reported savings from individual optimization studies are therefore informative but cannot automatically be generalized because the baseline operating strategy, plant configuration, and economic assumptions differ [101,102,103,104,109,110]. Regulatory acceptance represents an additional constraint. Water and wastewater facilities operate within environments where treatment reliability and public safety take precedence over marginal optimization gains. A regulator or plant operator may therefore prefer a slightly less optimal but transparent and predictable control strategy over a highly optimized black-box policy whose failure modes are poorly understood. This helps explain why many current applications remain at advisory Levels 1–2 of the autonomy framework rather than progressing directly toward fully autonomous control. Staged deployment, including laboratory validation, pilot demonstration, shadow operation, supervised control, and, finally, bounded automation, therefore provides a rational risk management pathway rather than merely reflecting technological immaturity [36,65,97].

### 12.7. Workforce and Multidisciplinary Capability

Finally, intelligent membrane systems require expertise spanning membrane science, process engineering, data science, control engineering, cybersecurity, and materials informatics. These capabilities are rarely concentrated within a single conventional plant engineering role. The resulting skills gap may become increasingly important as DTs evolve from analytical dashboards into continuously maintained cyber-physical systems. Training, interdisciplinary research teams, and shared digital infrastructure platforms will therefore be necessary to ensure that operators can understand, supervise, and challenge automated recommendations rather than becoming dependent on opaque systems. Overall, these limitations suggest that the central research challenge is not to develop increasingly complex ML algorithms in isolation. The challenge is to establish validated, interoperable, physically consistent, and economically justified cyber-physical systems that remain trustworthy when operating conditions change. The evidence therefore supports a staged progression: strengthen Level 2 predictive capabilities, validate narrowly scoped Level 3 closed-loop actions, expand toward Level 4 only where uncertainty and safety can be bounded, and reserve Level 5 design–operate–maintain autonomy for applications where the necessary technical, economic, and institutional foundations have been demonstrated.

## 13. Research and Deployment Roadmap

**Figure 11:**
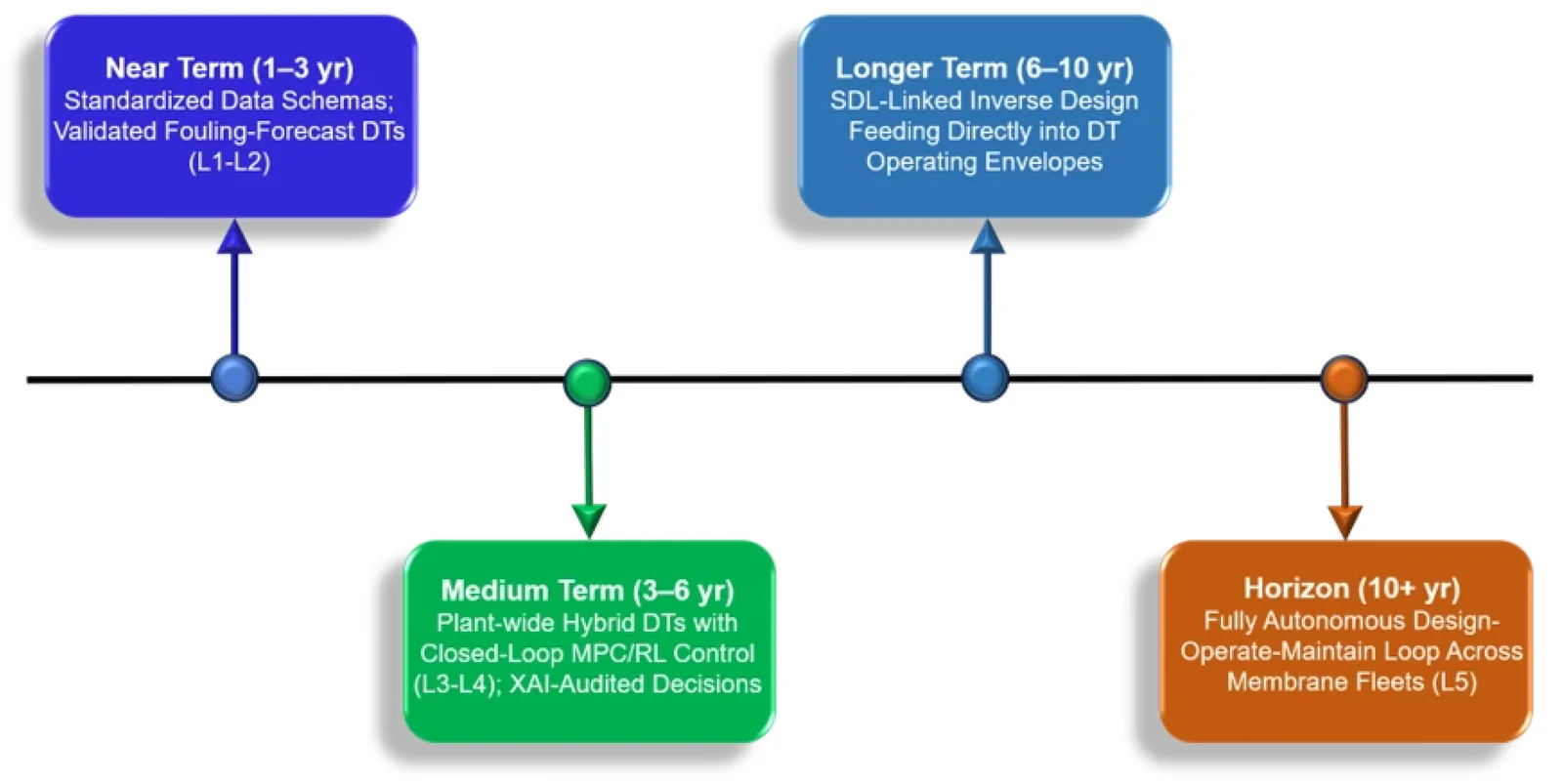
Research and deployment roadmap towards autonomous membrane systems.

Drawing together the evidence from this review, the transition toward intelligent and autonomous membrane systems should be pursued as a staged research-and-deployment program rather than as an immediate attempt to achieve full autonomy. The proposed roadmap in Figure 11 therefore links technical development to the five-level autonomy framework and prioritizes validation, interoperability, and trust before increasing decision authority. It should be noted that the time horizons in the roadmap are indicative and are intended to represent relative research maturity rather than guaranteed deployment dates.

Near term (1–3 years)—establish the data and validation foundation: The immediate priority should be to improve the reproducibility and generalizability of membrane ML and DT studies. Standardized data schemas, common variable definitions, shared ontologies, and consistent reporting of sampling frequency, missing data, sensor quality, and operating domains should be developed. Multi-site benchmark datasets or federated learning frameworks where data cannot be centrally shared should enable temporal, seasonal, and cross-plant validation rather than reliance on random within-site train/test splits. Autopsy-informed validation should also become more common for fouling diagnosis, providing independent physicochemical evidence against which model-attributed fouling mechanisms can be evaluated. During this period, most deployments should remain within Levels 1–2, emphasizing reliable monitoring, state estimation, forecasting, and explainable decision support.

Medium term (3–6 years)—validate bounded autonomous control: Once predictive models demonstrate external validity, research should progress toward Level 3 and selected Level 4 applications. The most appropriate initial targets are narrow, reversible, and readily monitored actions, including antiscalant/coagulant dosing, flux adjustment, backwashing, and CIP scheduling. RL and MPC policies currently demonstrated predominantly in simulation should undergo staged pilot and field validation, initially operating in shadow mode before receiving limited control authority. Physics-informed or hybrid DTs, uncertainty quantification, XAI, and automatic fallback strategies should be incorporated into these demonstrations. Cybersecurity-by-design should become a mandatory architectural component for any system capable of influencing physical actuators.

Long term (6–10 years)—connect operation with materials discovery: The most important structural opportunity identified by this review is the integration of operational DTs with autonomous membrane materials discovery. Shared data infrastructures should connect plant-level performance, fouling, degradation, energy, and maintenance information with materials informatics platforms. These data could define realistic performance requirements for inverse design algorithms, while self-driving laboratories could generate and experimentally validate candidate materials. Newly discovered membranes should subsequently be evaluated through process-level DTs under realistic operating conditions, creating a bidirectional connection between materials design and process operation [17,19,70,137].

The 10-year-plus horizon—develop the design–operate–maintain loop: Level 5 should be regarded as a technically plausible but conditional endpoint rather than an inevitable destination. In such a system, fleet-wide operational experience would continuously inform materials discovery, autonomous laboratories would generate and validate candidate membranes, and validated materials and operating strategies would feed back into plant DTs. Human involvement would shift from routine intervention toward supervision of objectives, safety constraints, governance, and exceptional events. Achieving this vision will require mature interoperability, robust cybersecurity, validated uncertainty-aware models, economically justified automation, and regulatory confidence. The resulting architecture would move membrane technology beyond isolated predictive models toward a continuously learning design–operate–maintain ecosystem while retaining human override and accountability wherever autonomous action presents unacceptable technical or societal risk.

## 14. Conclusions

This review examined the convergence of machine learning (ML), digital twins (DTs), and autonomous materials design across membrane technologies, framing them as stages in the progression toward increasingly autonomous membrane systems. ML has matured in fouling prediction, performance forecasting, diagnosis, and process optimization, while DTs provide the cyber-physical infrastructure needed to connect these capabilities with real-time membrane operation. In parallel, ML-assisted inverse design, generative modeling, Bayesian optimization, and self-driving laboratories are shifting membrane materials discovery from trial-and-error development toward computational–experimental closed-loop design. Three conclusions emerge. First, distinguishing digital models, digital shadows, and digital twins is essential for assessing DT maturity, since many systems labeled as DTs still require human intervention before recommendations reach physical processes. Second, the five-level autonomy framework provides a common basis for comparing applications ranging from assisted monitoring and fouling prediction to closed-loop control and autonomous materials discovery. Third, substantial opportunities exist in connecting operational and materials design intelligence. Data on fouling, degradation, energy use, cleaning, and membrane lifetime could guide materials discovery, while new membranes could be evaluated through operational DTs before deployment. However, higher autonomy depends on more than algorithmic sophistication. Data quality, generalizability, model drift, interoperability, cybersecurity, explainability, uncertainty, techno-economic feasibility, regulatory trust, and multidisciplinary expertise remain important barriers. A credible pathway is therefore staged: establish robust Level 1–2 predictive systems, validate targeted Level 3 control applications, progress cautiously toward Level 4 autonomy, and pursue Level 5 design–operate–maintain systems where technical and institutional conditions permit. Ultimately, intelligent membrane technology requires a validated, interoperable, continuously learning cyber-physical ecosystem connecting materials, operation, and maintenance across the lifecycle.

## Figures and Tables

**Table 1:** Search strings used for each thematic cluster.

Cluster	Search String
C1: Fouling AI/ML	TITLE-ABS-KEY((membrane OR “reverse osmosis” OR nanofiltration OR ultrafiltration OR microfiltration OR “membrane bioreactor” OR “membrane distillation”) AND (foul* OR scaling OR “transmembrane pressure” OR flux) AND (“machine learning” OR “artificial intelligence” OR “neural network*” OR “deep learning” OR “reinforcement learning” OR “random forest” OR “support vector”))
C2: DT in water/wastewater	TITLE-ABS-KEY((“digital twin*” OR “digital shadow*” OR “digital model*”) AND (water OR wastewater OR desalination OR “water treatment” OR membrane))
C3: Hybrid/DS/DT membranes	TITLE-ABS-KEY((“digital twin*” OR “digital shadow*” OR “hybrid model*” OR “physics-informed” OR “grey-box” OR “gray-box” OR “model predictive control”) AND (membrane* OR “reverse osmosis” OR “gas separation” OR pervaporation))
C4: Materials design	TITLE-ABS-KEY((membrane*) AND (“machine learning” OR “artificial intelligence” OR “inverse design” OR “generative” OR “Bayesian optimization*” OR “genetic algorithm” OR “self-driving lab*” OR “autonomous lab*” OR “high-throughput”) AND (design OR discovery OR screening OR “materials informatics”))

**Table 2:** Comparative positioning of the present review relative to major thematic clusters in the existing literature.

Dimension	C1: Fouling ML	C2: Water DT	C3: Hybrid/DS/DT	C4: Materials ML	Present Review
Primary focus	Fouling prediction, diagnosis, control	Water infrastructure digitalization	Physics–data integration, DT architectures	Property prediction, inverse design	Operation to autonomous design
DM–DS–DT distinction	Limited	Conceptual, selective	More prominent	Outside scope	Applied as classification criterion
Autonomy framework	Absent	Absent	Partial	Absent	Levels 0–5 (Section 10)
Real-time optimization	Limited–moderate	Moderate	Moderate–strong	Minimal	Linked to DT, MPC, and RL
Fouling mechanisms	Strong	Weak–moderate	Moderate–strong	Minimal	Grounded in DT state variables
Inverse design/self-driving labs	Absent	Absent	Rare	Central/increasing	Upper autonomy stage
Design–operation integration	Limited	Limited	Limited	Limited	Central: design–operate–maintain loop

**Table 3:** Representative ML methods by function in membrane systems.

Function	Representative Methods	Typical Inputs	Typical Outputs	Reported Evidence	Ref.(s)
State Estimation/Soft Sensing	Kalman/EnKF, hybrid physics–ML, PINN-coupled estimators	TMP, permeate flux, pressure, flow, conductivity, temperature, etc.	Membrane resistance, fouling state, permeability	Performance is highly case-specific; recursive estimation can improve robustness to noisy/incomplete measurements and model–plant mismatch.	[91,92]
Predictive Forecasting—Shallow ML	MLR, ANN, SVR, RF, XGBoost	TMP, flux, temperature, turbidity, pH, conductivity, flow rate, water quality, etc.	TMP, flux decline, fouling indicators	Strong performance has been reported in individual plants, but generalization varies considerably. A study reported test set R^2^ up to 0.435 for SVR, while other full-scale MBR studies report substantially higher values depending on dataset and validation design.	[14,29,94]
Predictive Forecasting—Deep/Sequence Models	LSTM, GRU, Seq2Seq, CNN–LSTM, Transformers	Multivariate time series histories of TMP, flux, pressure, flow, water quality, etc.	Multi-step TMP, fouling, flux, energy	Reported case studies include R^2^ ≈ 0.96–0.99 for selected GRU/LSTM configurations and R^2^ >0.87 in full-scale MBR forecasting; performance is strongly dependent on horizon, dataset, and validation strategy.	[26,95,100]
Diagnosis and Attribution	SHAP, LIME, permutation importance, partial dependence	Trained predictive model plus process and water quality features	Ranked drivers, local/global explanations	Primarily evaluated through interpretability and consistency with known fouling mechanisms rather than a single predictive metric. SHAP flagged F/M ratio, MLSSs, pH, and related temporal features as influential in MBR fouling prediction.	[45,47,96,97]
Prescriptive Decision-Making/Control	DQN, DDPG, RL, MPC–ML, BO, GA	Predicted process state, operating constraints, objective/cost functions, energy demand, and fouling indicators	Setpoints, dosing, backwash/CIP triggers	Evidence remains predominantly application- and simulation-specific. Reported studies demonstrate optimization of flux, fouling, and/or rejection, but plant-scale autonomous validation remains limited.	[11,34,98]
Materials Design/Inverse Design	GA, BO, VAEs, GNNs, forward/inverse models	Polymer structural descriptors, molecular representations, and physicochemical properties	Candidate polymers, predicted permeability/selectivity	Demonstrated primarily through computational screening and inverse design, experimental synthesis and validation remain important bottlenecks.	[17,41]

CNN—Convolutional Neural Network; DDPG—Deep Deterministic Policy Gradient; DQN—Deep Q-Network; EnKF—Ensemble Kalman Filter; GA—Genetic Algorithm; BO—Bayesian Optimization; GNN—Graph Neural Network; GRU—Gated Recurrent Unit; LSTM—Long Short-Term Memory; MLSSs—Mixed Liquor Suspended Solids; RF—Random Forest; RL—Reinforcement Learning; and VAE—Variational Autoencoder.

**Table 4:** Representative DT and hybrid model case studies.

Case Study Type	Process/Scale	DT Classification	Primary Function	Reported Outcome	Ref.
ANN fouling-phase model	Full-scale three-stage RO system, six years of operational data, 59 hydraulic and water quality variables, and 190 inter-cleaning runs	ML model	Identification of distinct fouling phases and their dominant predictors	Phase 1 was associated with particle deposition while Phase 2 involved gradual biofilm and scale growth. Different predictor sets were identified for each phase, providing a basis for targeted fouling mitigation and chlorine optimization.	[29]
LSTM encoder–decoder fouling forecaster embedded in ML-DT	Centralized MBR wastewater treatment plant, operational/process data	ML-enabled DT	Multi-step membrane-fouling forecasting and proactive cleaning support	LSTM encoder–decoder achieved MAPE < 6.45% and R^2^ > 0.87, supporting proactive decisions on online chemical cleaning under dynamic operating conditions.	[26]
Adaptive PINN + Ensemble Kalman Filter	Dynamic process system, real-world PMMA polymerization case	Adaptive DT (cross-domain exemplar)	Recursive state and parameter estimation under changing process conditions	Adaptive PINN–EnKF maintained predictive performance under parameter drift and model–plant mismatch; reported 10–20× accuracy improvement over non-adaptive DTs and approximately 96% lower computational time than physics-based EnKF.	[91]
Microservices-based DT platform	Municipal wastewater treatment plant; plant-wide architecture with edge computing capability	DT framework/architectural demonstrator	Integration of equipment, sensors, process models, ML, simulation, and decision support functions	Demonstrates modular integration of heterogeneous plant functions through microservices, supporting incremental development and edge deployment rather than requiring a monolithic DT architecture.	[87]
Deep-RL membrane cleaning controller	Laboratory-scale RO crossflow system with real-time fouling monitoring	Experimental/emerging DT control	Determine economically optimal cleaning intervention time	RL-based cleaning intervention reduced simulated cost per unit volume by 16%, although at the expense of approximately 9% lower permeate production. Later experimental deployment incorporated real-time fouling monitoring and operator interaction.	[119]
Deep-RL pressure and chemical-cleaning optimization	Laboratory-scale seawater UF system	Hybrid ML–RL control demonstrator	Simultaneously optimize operating pressure, cleaning time, and cleaning concentration	Reported an approximately 20.9% reduction in specific energy consumption, with the average flux increasing from 39.5 to 43.7 L m^−2^ h^−1^ while operating pressure decreased.	[117]
Deep-RL RO operational optimization	RO desalination, dynamic process-model environment	Simulation-based DT/RL controller	Joint optimization of plant operating cost, permeate flow, and water demand	Cascade DDPG–DQN architecture controlled the high-pressure pump and setpoints to reduce operating cost while maintaining required water production; long-term simulations incorporated membrane-fouling effects.	[105]
Multi-agent RL for two-stage RO	Industrial two-stage RO process represented using operational-data-calibrated numerical modeling	Simulation-based DT/emerging autonomous control framework	Optimize feed flow, high-pressure pump pressure, and inter-stage boosting pressure	VDN, QMIX, and DRQN were evaluated; multi-agent approaches outperformed the single-agent approach, with VDN achieving the lowest specific energy consumption while satisfying production requirements.	[106]

DDPG—Deep Deterministic Policy Gradient; DRQN—Deep Recurrent Q-Network; DQN—Deep Q-Network; PMMA—Poly(methyl methacrylate); QMIX—Monotonic Value Function Factorization for Deep Multi-Agent Reinforcement Learning; and VDN—Value Decomposition Network.

## Data Availability

No new data were created or analyzed in this study. Data sharing is not applicable to this article.

## References

[1] Khalil A.M. (2026). Recent Advances in Membrane Filtration and Purification Technologies. Membranes.

[2] Sharma R. (2026). Water Desalination Plant Market Research Report 2034. https://marketintelo.com/report/water-desalination-plant-market.

[3] Market Data Forecast. Membrane Bioreactor Market. https://www.marketdataforecast.com/market-reports/membrane-bioreactor-market.

[4] Usha Z.R., Liu C., Zhang S., Wang Z. (2025). A comprehensive study of recent advances, challenges and factors of nanofiltration membrane in various industrial applications. Desalination.

[5] Hou R., Fong C., Freeman B.D., Hill M.R., Xie Z. (2022). Current status and advances in membrane technology for carbon capture. Sep. Purif. Technol..

[6] Al-Harby N.F., El Batouti M., Elewa M.M. (2023). A Comparative Analysis of Pervaporation and Membrane Distillation Techniques for Desalination Utilising the Sweeping Air Methodology with Novel and Economical Pervaporation Membranes. Polymers.

[7] Park J.W., Heo S., Yeo J.-G., Lee S., Kim J.-K., Lee J.H. (2025). Membrane-Based CO_2_ Capture Across Industrial Sectors: Process Conditions, Case Studies, and Implementation Insights. Membranes.

[8] Nature Water Editorial (2026). Fundamentals governing membrane engineering. Nat. Water.

[9] Jiang S., Li Y., Ladewig B.P. (2017). A review of reverse osmosis membrane fouling and control strategies. Sci. Total Environ..

[10] Guo W., Ngo H.-H., Li J. (2012). A mini-review on membrane fouling. Bioresour. Technol..

[11] Bagheri M., Akbari A., Mirbagheri S.A. (2019). Advanced control of membrane fouling in filtration systems using artificial intelligence and machine learning techniques: A critical review. Process Saf. Environ. Prot..

[12] Shanmathi C., Hermanth Kumar K., Arthanareeswaran G., Thenmozhi K. (2025). Computational analysis of flux decline and fouling in ultrafiltration membranes using artificial neural network and hermia’s model. Chem. Eng. Res. Des..

[13] Nguyen T.N., Do K.-U., Tran T.P.N., Van Nguyen T., Chu Q.X. (2025). Predictive Modeling of Membrane Fouling in Domestic Wastewater Membrane Bioreactor Using Machine Learning. Water Environ. Res..

[14] Kovacs D.J., Li Z., Baetz B.W., Hong Y., Donnaz S., Zhao X., Zhou P., Ding H., Dong Q. (2022). Membrane fouling prediction and uncertainty analysis using machine learning: A wastewater treatment plant case study. J. Membr. Sci..

[15] Gümüş D., Alver A., Akbal F. (2026). Development of a digital twin framework for hybrid adsorption-ultrafiltration systems in drinking water treatment. Process Saf. Environ. Prot..

[16] Zhu Y., Wang Y., Zhu E., Ma Z., Wang H., Chen C., Guan J., Waite T.D. (2025). Predicting Membrane Fouling of Submerged Membrane Bioreactor Wastewater Treatment Plants Using Machine Learning. Environ. Sci. Technol..

[17] Dangayach R., Jeong N., Demirel E., Uzal N., Fung V., Chen Y. (2024). Machine Learning-Aided Inverse Design and Discovery of Novel Polymeric Materials for Membrane Separation. Environ. Sci. Technol..

[18] Chen L., Liu G., Zhang Z., Wang Y., Yang Y., Li J. (2024). Machine learning and molecular design algorithm assisted discovery of gas separation membranes exceeding the CO_2_/CH_4_ and CO_2_/N_2_ upper bounds. Chem. Eng. Sci..

[19] Ochiai H., Nagai K., Kaneko H. (2025). Machine Learning-Driven Design of Sustainable Polymer Membranes: Integrated Prediction of Gas Permeability, Selectivity, and Biodegradability. ACS Omega.

[20] Grieves M., Vickers J. (2016). Digital twin: Mitigating unpredictable, undesirable emergent behavior in complex systems. Transdisciplinary Perspectives on Complex Systems: New Findings and Approaches.

[21] Kritzinger W., Karner M., Traar G., Henjes J., Sihn W. (2018). Digital Twin in manufacturing: A categorical literature review and classification. IFAC Pap..

[22] Iossa R., Domenighini P., Cotana F. (2025). Digital Twins from Building to Urban Areas: An Open Opportunity to Energy, Environmental, Economic and Social Benefits. Appl. Sci..

[23] Singh M., Fuenmayor E., Hinchy E.P., Qiao Y., Murray N., Devine D. (2021). Digital Twin: Origin to Future. Appl. Syst. Innov..

[24] Wang A.-J., Li H., He Z., Tao Y., Wang H., Yang M., Savic D., Daigger G.T., Ren N. (2024). Digital Twins for Wastewater Treatment: A Technical Review. Engineering.

[25] van Rooij F., Scarf P., Do P. (2021). Planning the restoration of membranes in RO desalination using a digital twin. Desalination.

[26] Ma Z., Zhu Y., Chen C., Li T., Li Y., Li X., Wang Y., Waite T.D., Guan J. (2025). Towards the digitalization of water treatment facilities: A case study on machine learning-enabled digital twins. J. Water Process Eng..

[27] Ahanger T.A., Abdibayev Z., Sagnayeva S., Zhumadillayeva A., Dyussekeyev K. (2026). Smart wastewater management in hydro-technical systems using digital twin technology. Sci. Rep..

[28] Frick N., Terwolbeck J., Seibel B., Metternich J. (2024). Design Model for the Digital Shadow of a Value Stream. Systems.

[29] Roehl E.A., Ladner D.A., Daamen R.C., Cook J.B., Safarik J., Phipps D.W., Xie P. (2018). Modeling fouling in a large RO system with artificial neural networks. J. Membr. Sci..

[30] Dologlu P., Sildir H. (2022). Data driven identification of industrial reverse osmosis membrane process. Comput. Chem. Eng..

[31] Niu C., Li X., Dai R., Wang Z. (2022). Artificial intelligence-incorporated membrane fouling prediction for membrane-based processes in the past 20 years: A critical review. Water Res..

[32] Al-Kathiri D., Rao G.B., Qahoor N.M.S., Banerjee S., Babu N.R., Kavitha G., Lakkimsetty N.R., Namdeti R. (2025). Membrane fouling prediction and control using AI and machine learning: A comprehensive review. J. Environ. Earth Sci..

[33] Yan X., Liu C., Bao R., Gao C., Ma C., Kong X. (2026). Prediction of membrane fouling types in membrane distillation from digital microscope images based on a CNN model. Sep. Purif. Technol..

[34] Yang X., Zhao J., Zhuang J., Guan S., Wang C., Huang K., Liu L., Gao Y. (2026). Mechanism-aligned machine learning for membrane fouling prediction and process control in pressure-driven water treatment membranes: A review. J. Water Process Eng..

[35] Garakani S.S., Chew J.W. (2025). Development of physics-informed machine-learning models to enhance understanding and prediction of membrane fouling. J. Membr. Sci..

[36] Ghasemzadeh K., Jafari M., Torabi T., Amiri A., Iulianelli A. (2026). Hybrid models, digital twins, and digital shadows for sustainable membrane technologies: A critical review. Adv. Membr..

[37] Raissi M., Perdikaris P., Karniadakis G.E. (2019). Physics-informed neural networks: A deep learning framework for solving forward and inverse problems involving nonlinear partial differential equations. J. Comput. Phys..

[38] Gaublomme D., Quaghebeur W., Van Droogenbroeck A., Vanoppen M., De Gusseme B., Verliefde A., Nopens I., Torfs E. (2023). A hybrid modelling approach for reverse osmosis processes including fouling. Desalination.

[39] Møller J.K., Goranović G., Brath P., Madsen H. (2022). A data-driven digital twin for water ultrafiltration. Commun. Eng..

[40] Heusel M., Grim G., Rauhut J., Franzreb M. (2024). Regression Metamodel-Based Digital Twin for an Industrial Dynamic Crossflow Filtration Process. Bioengineering.

[41] Basdogan Y., Pollard D.R., Shastry T., Carbone M.R., Kumar S.K., Wang Z.-G. (2024). Machine learning-guided discovery of polymer membranes for CO_2_ separation with genetic algorithm. J. Membr. Sci..

[42] Yang J., Tao L., He J., McCutcheon J.R., Li Y. (2022). Machine learning enables interpretable discovery of innovative polymers for gas separation membranes. Sci. Adv..

[43] Volk A.A., Epps R.W., Yonemoto D.T., Masters B.S., Castellano F.N., Reyes K.G., Abolhasani M. (2023). AlphaFlow: Autonomous discovery and optimization of multi-step chemistry using a self-driven fluidic lab guided by reinforcement learning. Nat. Commun..

[44] Mahmoud M., Lazarova-Molnar S. Explainability in Digital Twins: Overview and Challenges. Proceedings of the 2025 Winter Simulation Conference (WSC).

[45] Al-Rashed W.S. (2026). Wastewater Membrane Bioreactors: A Comprehensive Review of Explainable Artificial Intelligence and Digital Twin Applications. Membranes.

[46] Talhami M., Wakjira T., Alomar T., Fouladi S., Fezouni F., Ebead U., Altaee A., Al-Ejji M., Das P., Hawari A.H. (2024). Single and ensemble explainable machine learning-based prediction of membrane flux in the reverse osmosis process. J. Water Process Eng..

[47] Liang J., Lee S., Ren X., Guo Y., Park J., Park S.-G., Kim J.-Y., Hwang M.-H. (2025). Predictive Framework for Membrane Fouling in Full-Scale Membrane Bioreactors (MBRs): Integrating AI-Driven Feature Engineering and Explainable AI (XAI). Processes.

[48] Song Y., Moon J., Park K. (2025). Explainable AI for permeate flux prediction in forward osmosis: SHAP interpretability and theoretical validation for enhanced predictive reliability. Desalination.

[49] Lam C.F.D., van Moorsel A., Pourmirza Z. (2026). Trustworthiness in Digital Twin Systems: Systematic Review and Research Horizons. arXiv.

[50] Jawad J., Hawari A.H., Zaidi S.J. (2021). Artificial neural network modeling of wastewater treatment and desalination using membrane processes: A review. Chem. Eng. J..

[51] Gaudio M.T., Coppola G., Zangari L., Curcio S., Greco S., Chakraborty S. (2021). Artificial intelligence-based optimization of industrial membrane processes. Earth Syst. Environ..

[52] Viet N.D., Jang D., Yoon Y., Jang A. (2022). Enhancement of membrane system performance using artificial intelligence technologies for sustainable water and wastewater treatment: A critical review. Crit. Rev. Environ. Sci. Technol..

[53] AlSawaftah N., Abuwatfa W., Darwish N., Husseini G.A. (2022). A review on membrane biofouling: Prediction, characterization, and mitigation. Membranes.

[54] Ray S.S., Verma R.K., Singh A., Ganesapillai M., Kwon Y.-N. (2023). A holistic review on how artificial intelligence has redefined water treatment and seawater desalination processes. Desalination.

[55] Abuwatfa W.H., AlSawaftah N., Darwish N., Pitt W.G., Husseini G.A. (2023). A Review on Membrane Fouling Prediction Using Artificial Neural Networks (ANNs). Membranes.

[56] Frontistis Z., Lykogiannis G., Sarmpanis A. (2023). Machine learning implementation in membrane bioreactor systems: Progress, challenges, and future perspectives: A review. Environments.

[57] Wang L., Li Z., Fan J., Han Z. (2024). The intelligent prediction of membrane fouling during membrane filtration by mathematical models and artificial intelligence models. Chemosphere.

[58] Cairone S., Hasan S.W., Choo K.-H., Li C.-W., Zorpas A.A., Ksibi M., Zarra T., Belgiorno V., Naddeo V. (2024). Enhancing membrane fouling control in wastewater treatment processes through artificial intelligence modeling: Research progress and future perspectives. Euro-Mediterr. J. Environ. Integr..

[59] Abdulsalam A., Le T.P.P., Nguyen T.S., Ali J.K., Abubasha M.Y., Jouini M.S., Alhseinat E. (2025). A review of fouling prediction techniques in membrane desalination: From empirical to intelligent models. Desalination.

[60] Abuhabib A., Albahnasavi A., Isawi H., Ng L.Y., Mohammad A.W., Hamzah S. (2026). Applying machine learning and AI for nanofiltration membranes water applications: A review. Water Sci. Technol..

[61] Dansawad P., Li Y., Li Y., Zhang J., You S., Li W., Yi S. (2023). Machine learning toward improving the performance of membrane-based wastewater treatment: A review. Adv. Membr..

[62] Liang Y.Y., Li M. (2025). Unsteady-state dynamics and AI in membrane desalination: Challenges and emerging opportunities. Desalination.

[63] Ghorbani Bam P., Rezaei N., Roubanis A., Austin D., Austin E., Tarroja B., Takacs I., Villez K., Rosso D. (2025). Digital twin applications in the water sector: A review. Water.

[64] Qureshi M., Feng Z., Kumar V., Besharat M., Lovreglio R. (2026). A systematic literature review of digital twin applications for sustainable water systems in natural and built environment. Discov. Sustain..

[65] Nikou N.S.R., Gill L., Ghaani M.R. (2026). Digital twins in wastewater treatment: A review of multiscale applications for process optimization, resource recovery, and policy compliance. J. Water Process Eng..

[66] Omar N.M.A., Amhamed A.O.A., Zhmoul N.S.E., Alhallak L.M., Krir N.A.M., Milad M. (2026). Digital twin-assisted next-generation ceramic membranes for sustainable heavy metal remediation. Chem. Eng. Process.-Process Intensif..

[67] Pappas V.M. (2026). From Observability to Intervention in Purification: A Critical Framework for Digital Twins, Chemometrics, and Control Across Membranes, Adsorption, and Chromatography. Purification.

[68] Talukder M.J., Alshami A.S., Tayyebi A., Ismail N., Yu X. (2024). Membrane science meets machine learning: Future and potential use in assisting membrane material design and fabrication. Sep. Purif. Rev..

[69] Yin H., Xu M., Luo Z., Bi X., Li J., Zhang S., Wang X. (2024). Machine learning for membrane design and discovery. Green Energy Environ..

[70] Cao Z., Barati Farimani O., Ock J., Barati Farimani A. (2024). Machine learning in membrane design: From property prediction to AI-guided optimization. Nano Lett..

[71] Xu J., Suleiman A., Liu G., Zhang R., Jiang M., Guo R., Luo T. (2024). Transcend the boundaries: Machine learning for designing polymeric membrane materials for gas separation. Chem. Phys. Rev..

[72] Osman A.I., Nasr M., Farghali M., Bakr S.S., Eltaweil A.S., Rashwan A.K., Abd El-Monaem E.M. (2024). Machine learning for membrane design in energy production, gas separation, and water treatment: A review. Environ. Chem. Lett..

[73] Ignacz G., Bader L., Beke A.K., Ghunaim Y., Shastry T., Vovusha H., Carbone M.R., Ghanem B., Szekely G. (2025). Machine learning for the advancement of membrane science and technology: A critical review. J. Membr. Sci..

[74] Meng C., Zhu H., Ghoufi A. (2025). Machine learning-driven mass transfer modeling and inverse design for nanofiltration membranes: A review. Desalination.

[75] Bora L.V. (2025). Leveraging the benefits of machine learning in membrane technology: A state-of-the-art review. Inorg. Chem. Commun..

[76] Liang L., Lu D., Qin Y., Chen J., Zhang L. (2025). Machine learning in membrane science: Bridging materials, structures, and performance for next-generation membrane design. Sep. Purif. Technol..

[77] Nazari S., Abdelrasoul A. (2025). Advancements and applications of artificial intelligence and machine learning in material science and membrane technology: A comprehensive review. Membranes.

[78] Jafari M., Tzirtzipi C., Castro-Dominguez B. (2024). Applications of artificial intelligence for membrane separation: A review. J. Water Process Eng..

[79] Chen J., Yang Y.-C., Huang J.-Y. (2013). On-line monitoring and diagnosis of membrane fouling using ultrasonic techniques. Chemom. Intell. Lab. Syst..

[80] Alshraideh H., Al Ali F., Awad M., Al-Othman A. (2026). AI-based remaining useful life prediction of seawater desalination membranes. Chem. Eng. J. Adv..

[81] Willcox K., Bingham D., Chung C., Chung J., Cruz-Neira C., Grant C.J., Kinter J., Leung R., Moin P., Ohno-Machado L. (2024). Foundational Research Gaps and Future Directions for Digital Twins.

[82] Somers R.J., Douthwaite J.A., Wagg D.J., Walkinshaw N., Hierons R.M. (2023). Digital-twin-based testing for cyber–physical systems: A systematic literature review. Inf. Softw. Technol..

[83] Yang M., Chen K., Gui N., Tao Y., Chen Y., Liu J., De Baets B., Wang A. (2026). Toward a Digital Twin for Industrial Wastewater Treatment Plants: A Framework Integrating Mechanistic and Deep Learning Models. Engineering.

[84] (2021). Automation Systems and Integration—Digital Twin Framework for Manufacturing—Part 1: Overview and General Principles, 1st ed.

[85] (2021). Automation Systems and Integration: Digital Twin Framework for Manufacturing: Part 2: Reference Architecture, 1st ed.

[86] (2021). Automation Systems and Integration—Digital Twin Framework for Manufacturing Part 4: Information Exchange, 1st ed.

[87] Rodríguez-Alonso C., Pena-Regueiro I., García Ó. (2024). Digital Twin Platform for Water Treatment Plants Using Microservices Architecture. Sensors.

[88] (2021). Automation Systems and Integration—Digital Twin Framework for Manufacturing Part 5: Digital Thread for Digital Twin, 1st ed.

[89] (2021). Automation Systems and Integration—Digital Twin Framework for Manufacturing Part 6: Digital Twin Composition, 1st ed.

[90] Chen F., Long J., Du Z., Song M., Liu X., Deng J., Cheng K., Huang Q. (2026). Joint estimation method of state and parameter of digital twin model based on adaptive ensemble kalman particle filter. Energy.

[91] Thosar D., Bhakte A., Li Z., Srinivasan R., Prasad V. (2026). Adaptive physics-informed neural network-based digital twins integrated with Ensemble Kalman Filter. Comput. Chem. Eng..

[92] Kim D.Y., Lee M.H., Lee S., Kim J.H., Yang D.R. (2009). Online estimation of fouling development for SWRO system using real data. Desalination.

[93] Taylor R., Mandur J., Amira U., Al-Inati N., Marin-Celis J., Hatch S., Cerezo L.F., Pinto N., Metsi-Guckel E., Matos T. (2025). Use of live digital twin (shadow) soft sensor to monitor membrane degradation in continuous manufacturing single pass tangential flow filtration. Biotechnol. Prog..

[94] Kherraf S., Abahdou F.-Z., Benbouzid M., Izouaouen Z., Aarfane A., Baraket A., Nasrellah H., Bensemlali M., Ziti S., Labjar N. (2026). Prediction of Reverse Osmosis Membrane Fouling Using Machine Learning: MLR, ANN, and SVM at a Seawater Desalination Plant. Eng.

[95] Moon J., Jeong K., Chae S.H., Shim J., Kim J., Cho K.H., Park K. (2025). Robust deep learning model combined with missing input data estimation: Application in a 1000 m^3^/day high-salinity SWRO plant. Desalination.

[96] Han X., Yang L., Qin H., Huang S., Zhang H., Xu B., Ng H.Y. (2026). Explainable cross-plant transfer learning for fouling prediction in data-limited pilot-scale membrane bioreactors. J. Membr. Sci..

[97] Zhu M., Gao S., Chen Y., Li S., Meng J., Yang J., Li N., Lu Y., Zhu G. (2026). AI for membrane bioreactors: Fouling prediction and decision support. J. Water Process Eng..

[98] Mubashir M., Mustakeem M., Alnumani A., Abutaleb A., Sumayli A.H.N., Ahmad T., Azhar M.R. (2025). Smart and Sustainable Regeneration of Fouled Desalination Membranes Using Artificial Intelligence. Glob. Chall..

[99] Croll H.C., Ikuma K., Ong S.K., Sarkar S. (2023). Reinforcement learning applied to wastewater treatment process control optimization: Approaches, challenges, and path forward. Crit. Rev. Environ. Sci. Technol..

[100] Yan C., Yalin N.I.U., Xing P., Wenjing Z., Xiaohu X.U., Pengcheng X.U. (2025). Prediction of Membrane Fouling in Pilot Nanofiltration System based on Deep Learning Model. J. Lanzhou Jiaotong Univ..

[101] Abdullah F., Villez K., Allu S. (2025). Machine learning modeling and model predictive control of a closed-circuit reverse osmosis system. Chem. Eng. Res. Des..

[102] Gao L., Jarma Y.A., Christofides P.D., Cohen Y. (2025). Real-Time Energy Optimal Control of Two-Stage Reverse Osmosis Desalination. Water.

[103] Gao L., Rahardianto A., Gu H., Christofides P.D., Cohen Y. (2013). Energy-Optimal Control of RO Desalination. Ind. Eng. Chem. Res..

[104] Nurjanah I., Chiang Y.-H., Jamaludin M., Sean W.-Y. (2025). Development of adaptive control algorithm with optimizing energy consumption verified by reverse osmosis process. Sep. Purif. Technol..

[105] Golabi A., Erradi A., Qiblawey H., Tantawy A., Bensaid A., Shaban K. (2024). Optimal operation of reverse osmosis desalination process with deep reinforcement learning methods. Appl. Intell..

[106] Yun B., Shim J., Moon J., Lee S., Jeong K., Cho K.H. (2025). Towards autonomous operation of two-stage reverse osmosis water treatment system with multi-agent reinforcement learning. Desalination.

[107] Al-Hamzawi H.A.H., Al Sailawi A.S.A. (2026). Multi-objective optimization of hybrid MSF–RO desalination systems under dynamic operating conditions using thermodynamics-guided surrogate-assisted reinforcement learning. Chem. Eng. Res. Des..

[108] Moon J., Yun B., Park K., Kim S.-S., Lee Y., Jeong K., Cho K.H. (2026). Adaptive reinforcement learning for energy-efficient high-recovery closed-circuit reverse osmosis. Water Res..

[109] Ridwan M.G., Altmann T., Yousry A., Das R. (2023). Intelligent framework for coagulant dosing optimization in an industrial-scale seawater reverse osmosis desalination plant. Mach. Learn. Appl..

[110] Mangal M.N., Yangali-Quintanilla V.A., Salinas-Rodriguez S.G., Dusseldorp J., Blankert B., Kemperman A.J.B., Schippers J.C., Kennedy M.D., van der Meer W.G.J. (2022). Application of a smart dosing pump algorithm in identifying real-time optimum dose of antiscalant in reverse osmosis systems. J. Membr. Sci..

[111] García-Triñanes P., Chairopoulou M.A., Campos L.C. (2022). Investigating reverse osmosis membrane fouling and scaling by membrane autopsy of a bench scale device. Environ. Technol..

[112] Ranzani da Costa T., Peter Schneider R. (2019). Critical evaluation of procedures fundamental to reverse osmosis membrane autopsy. Desalin. Water Treat..

[113] Tang F., Hu H.-Y., Sun L.-J., Wu Q.-Y., Jiang Y.-M., Guan Y.-T., Huang J.-J. (2014). Fouling of reverse osmosis membrane for municipal wastewater reclamation: Autopsy results from a full-scale plant. Desalination.

[114] Zheng L., Yu D., Wang G., Yue Z., Zhang C., Wang Y., Zhang J., Wang J., Liang G., Wei Y. (2018). Characteristics and formation mechanism of membrane fouling in a full-scale RO wastewater reclamation process: Membrane autopsy and fouling characterization. J. Membr. Sci..

[115] Tran T., Bolto B., Gray S., Hoang M., Ostarcevic E. (2007). An autopsy study of a fouled reverse osmosis membrane element used in a brackish water treatment plant. Water Res..

[116] Shim J., Lee S., Park S., Moon J., Lee C., Cho K.H. (2025). Optimizing membrane cleaning strategy of industrial reverse osmosis process using long sequence time-series forecasting. Desalination.

[117] Park S., Shim J., Yoon N., Lee S., Kwak D., Lee S., Kim Y.M., Son M., Cho K.H. (2022). Deep reinforcement learning in an ultrafiltration system: Optimizing operating pressure and chemical cleaning conditions. Chemosphere.

[118] Zhao B., Chen H., Gao D., Xu L., Zhang Y. (2020). Cleaning decision model of MBR membrane based on Bandelet neural network optimized by improved Bat algorithm. Appl. Soft Comput..

[119] Glover T. (2023). The Development of Real-Time Fouling Monitoring and Control Systems for Reverse Osmosis Membrane Cleaning using Deep Reinforcement Learning. Ph.D. Dissertation.

[120] Ramkumar N., Chandran D.G., Monash P. (2026). AI-ML innovations in membrane technology: Current progress and future prospects. Chem. Eng. J. Adv..

[121] Fan J., Zhang C., Gu K., Liu R., Qin Y., Laaksonen A., Zhu Y., Ji X., Lu X. (2026). Physics-Guided Machine Learning with Roughness-Corrected Interfacial Descriptors for Membrane Fouling Prediction. Ind. Eng. Chem. Res..

[122] Li M., Li J. (2025). Physics-informed neural networks for modeling and diagnosing degradation in reverse osmosis membranes. Desalin. Water Treat..

[123] Garakani S.S., Hassanabadi M., Chew J.W. (2026). A physics-informed neural network for interpretable membrane-fouling prediction with adaptive transitions between fouling mechanisms. Sep. Purif. Technol..

[124] Peng C., Li B., Yang J., Zhu J. (2026). Artificial Intelligence in Digital Twins: Paradigm, Enabling Technologies and Advantages. Digit. Twins Appl..

[125] Galinha C.F., Crespo J.G. (2021). From Black Box to Machine Learning: A Journey through Membrane Process Modelling. Membranes.

[126] Nele L., Mattera G., Yap E.W., Vozza M., Vespoli S. (2024). Towards the application of machine learning in digital twin technology: A multi-scale review. Discov. Appl. Sci..

[127] Ayton G.S., Izvekov S., Noid W.G., Voth G.A., Feller S.E. (2008). Chapter 7 Multiscale Simulation of Membranes and Membrane Proteins: Connecting Molecular Interactions to Mesoscopic Behavior. Current Topics in Membranes.

[128] Binger Z.M., Achilli A. (2023). Surrogate modeling of pressure loss & mass transfer in membrane channels via coupling of computational fluid dynamics and machine learning. Desalination.

[129] Wang M., Wang S., Lin J., Luo K., Fan J. (2026). Multi-scale computations of reactive multiphase flows: Towards digital twins for energy transition. Particuology.

[130] Wu H., Wang X., Su W., Yang S., Li Z. (2026). Machine Learning-based Reduced-Order Model and multi-fidelity approaches for field reconstruction in CFD: A review. Chem. Eng. Sci..

[131] Tom G., Schmid S.P., Baird S.G., Cao Y., Darvish K., Hao H., Lo S., Pablo-García S., Rajaonson E.M., Skreta M. (2024). Self-Driving Laboratories for Chemistry and Materials Science. Chem. Rev..

[132] Hysmith H., Foadian E., Padhy S.P., Kalinin S.V., Moore R.G., Ovchinnikova O.S., Ahmadi M. (2024). The future of self-driving laboratories: From human in the loop interactive AI to gamification. Digit. Discov..

[133] Canty R.B., Abolhasani M. (2026). The past, present and future of self-driving laboratories. Nat. Rev. Chem..

[134] Low A.K.Y., Cheng J.J.W., Hippalgaonkar K., Ng L.W.T. (2025). Self-Driving Laboratories: Translating Materials Science from Laboratory to Factory. ACS Omega.

[135] Phan B.K., Shen K.-H., Gurnani R., Tran H., Lively R., Ramprasad R. (2024). Gas permeability, diffusivity, and solubility in polymers: Simulation-experiment data fusion and multi-task machine learning. npj Comput. Mater..

[136] Xu J., Suleiman A., Liu G., Perez M., Zhang R., Jiang M., Guo R., Luo T. (2024). Superior polymeric gas separation membrane designed by explainable graph machine learning. Cell Rep. Phys. Sci..

[137] Cheng M., Fu C.-L., Okabe R., Chotrattanapituk A., Boonkird A., Hung N.T., Li M. (2026). Artificial intelligence-driven approaches for materials design and discovery. Nat. Mater..

[138] Bonny T., Kashkash M., Ahmed F. (2022). An efficient deep reinforcement machine learning-based control reverse osmosis system for water desalination. Desalination.

